# Bayesian Estimation of Past Population Dynamics in BEAST 1.10 Using the Skygrid Coalescent Model

**DOI:** 10.1093/molbev/msz172

**Published:** 2019-07-31

**Authors:** Verity Hill, Guy Baele

**Affiliations:** 1 Institute of Evolutionary Biology, University of Edinburgh, Edinburgh, United Kingdom; 2 Department of Microbiology, Immunology and Transplantation, Rega Institute, KU Leuven, Leuven, Belgium

**Keywords:** BEAST, Skygrid, coalescent, Bayesian phylogenetics, pathogen phylodynamics, TempEst, Tracer

## Abstract

Inferring past population dynamics over time from heterochronous molecular sequence data is often achieved using the Bayesian Skygrid model, a nonparametric coalescent model that estimates the effective population size over time. Available in BEAST, a cross-platform program for Bayesian analysis of molecular sequences using Markov chain Monte Carlo, this coalescent model is often estimated in conjunction with a molecular clock model to produce time-stamped phylogenetic trees. We here provide a practical guide to using BEAST and its accompanying applications for the purpose of drawing inference under these models. We focus on best practices, potential pitfalls, and recommendations that can be generalized to other software packages for Bayesian inference. This protocol shows how to use TempEst, BEAUti, and BEAST 1.10 (http://beast.community/; last accessed July 29, 2019), LogCombiner as well as Tracer in a complete workflow.

## Introduction

The Bayesian Evolutionary Analysis by Sampling Trees (BEAST) software package (Suchard et al. 2018) allows the estimation of time-stamped phylogenetic trees from genetic data sampled at different time points using Markov chain Monte Carlo (MCMC) integration. Such inferences can be performed if the genetic sequences constitute a measurably evolving population (MEP; Drummond et al. 2003; Biek et al. 2015), meaning that they have undergone measurable amounts of evolutionary change between sampling times. Importantly, MEPs are not restricted to RNA viruses, as the field of ancient DNA research (see Supplementary Material online) also allows the estimation of divergence times and temporal changes in population size without additional calibrations (Drummond et al. 2003). When the assumptions of an MEP are fulfilled, a coalescent demographic model with a molecular clock model can be used in BEAST to estimate the effective population size over time.

Here, we focus on estimating changes in effective population size over time, which constitutes a frequently performed analysis on fast-evolving pathogens, for example for the reconstruction of early HIV-1 dynamics in the Democratic Republic of Congo (Faria et al. 2014) and the rise and decline of Ebola virus during the 2013–2016 West African epidemic (Dudas et al. 2017). One of the most important parameters in population genetics, effective population size translates the census size of a real population into the size of an idealized population showing the same rate of loss of genetic diversity as the real population under study (Husemann et al. 2016). It is therefore an abstract quantity which provides a measure of population size from genetic diversity under an idealized reproductive model which, when measured over time, provides an estimate of past population dynamics (Gill et al. 2013).

**Fig. 1.**
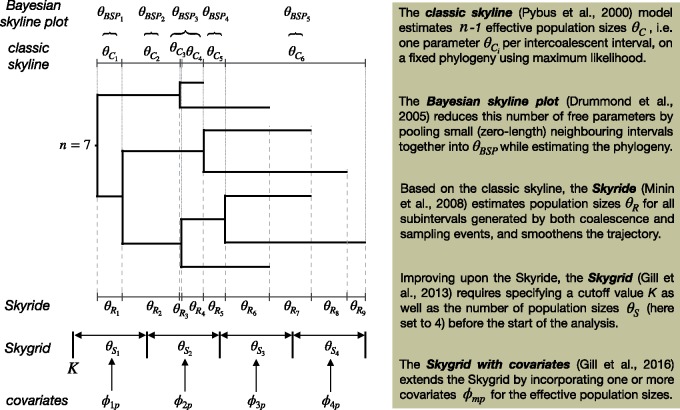
Conceptual representation of various nonparametric coalescent models on a phylogeny of *n *=* *7 heterochronous sequences. The classic skyline (Pybus et al. 2000) and its extension, the generalized skyline (Strimmer and Pybus 2001), were the first among a still increasing collection of nonparametric coalescent models. Initially estimated using maximum likelihood inference on a fixed phylogeny, these models have been extended for use in Bayesian framework while accommodating phylogenetic uncertainty (Drummond et al. 2005). Recent developments include the Skyride (Minin et al. 2008), the Skygrid (Gill et al. 2013), and its extension to incorporate covariates (Gill et al. 2016), which all employ smoothing priors.

Until two decades ago, only simple parametric coalescent models—such as exponential growth and constant population size models—were available, but their estimated demographic parameters are only considered meaningful if there is a prior reason to believe that the sampled population fits the specified demographic model (Pybus et al. 2000). Given that this is usually not the case, flexible nonparametric coalescent models have been developed that enable the estimation of a varying effective population size over time, allowing accurate estimation of population trajectories (see fig. 1).

The classic skyline (Pybus et al. 2000) was the first such model, estimating the population size trajectory on a fixed underlying phylogeny using maximum likelihood (ML). For a data set of *n* sequences, the classic skyline considers all *n*−1 intercoalescent intervals (i.e. the intervals on a phylogeny that separate the coalescence events), leading to potentially “noisy” estimates for the population size trajectory when the product of interval length and evolutionary rate is small (e.g. $\theta_{C_{3}}$ in fig. 1). The generalized skyline (Strimmer and Pybus 2001) reduced this noise by allowing multiple coalescence events (for which little divergence time information is available) to be grouped together (e.g. $\theta_{C_{3}}$ and $\theta_{C_{4}}$ in fig. 1), a process that is governed through an automated model selection procedure. Drummond et al. (2005) in turn extended this model to accommodate phylogenetic uncertainty in a Bayesian framework, yielding credibility intervals for the estimated effective population sizes.

Using a Gaussian Markov random field smoothing prior, the Skyride model (Minin et al. 2008) produces a temporal smoothing along the population trajectory by considering all coalescent events and sampling times ($\theta_{R_{1}}$–$\theta_{R_{9}}$ in fig. 1). The Skygrid (Gill et al. 2013) improves upon the Skyride by allowing the effective population size $\theta_{S_{i}}$ to change at user-defined points in real time, in addition to yielding improved root height estimation and allowing for estimation based on multilocus data. This model balances complexity with the ability to estimate a population size for each meaningful time interval, such as an epidemiological week during an outbreak. The Skygrid’s parameterization also paves the way for further extensions, such as the inclusion of external covariates $\varphi_{\mathrm{ij}}$ (Gill et al. 2016), enabling the examination of the impact of nongenetic factors on effective population size estimates.

In this protocol, we focus on the Skygrid model and provide a complete workflow (see fig. S1, Supplementary Material online) for estimating effective population sizes over time using this model in BEAST v1.10.4. We will be using other applications typically associated with the BEAST software package such as BEAUti (Bayesian Evolutionary Analysis Utility), TempEst (Temporal Exploration of Sequences and Trees) v1.5.3 (Rambaut et al. 2016), BEAGLE v3.1.0 (Ayres et al. 2019), LogCombiner, and Tracer v1.7.1 (Rambaut et al. 2018). All of these packages can be downloaded from the BEAST website (https://beast.community; last accessed July 29, 2019). We refer to Supplementary Material online for detailed information on aspects of this protocol not directly related to the Skygrid model, along with larger versions of the figures presented here.

We assume some prior knowledge of molecular phylogenetics and Bayesian inference when attempting this protocol. An intuitive explanation of the major concepts and features of Bayesian phylogenetic inference is provided by Huelsenbeck et al. (2001) and Nascimento et al. (2017), which also includes a discussion on prior specification, model choice, and data partitioning. We also recommend Volz et al. (2013) for a primer on molecular phylogenetics with specific reference to viruses, including a discussion of the main features of phylogenies that are important for identifying epidemiological, immunological, and evolutionary processes which influence patterns of genetic variation.

### Data Set Description

We construct a data set containing the first 200 Ebola virus sequences from Sierra Leone during the 2013–2016 West African Ebola virus epidemic. These sequences were sampled between June 26, 2014 and August 25, 2014 and have been previously published in Bell et al. (2015); Carroll et al. (2015); van Vuren et al. (2019); Gire et al. (2014) and Park et al. (2015). In collating the sequence data, we make sure that the sequence labels are all in the same format—with different fields separated by the same prefix, in this case the pipe/bar symbol **|** —and contain the sampling times using a consistent date format, here **year–month–day**, which simplifies the various steps in this protocol. We align the collected data using MAFFT (Nakamura et al. 2018), which we manually postprocess in Geneious 11.0.5 (https://www.geneious.com; last accessed July 29, 2019; see Supplementary Material online). Geneious is a commercial application but other alignment editors are freely available, such as MEGA (Kumar et al. 2012) and AliView (Larsson 2014).

We accommodate the specific features of our alignment by creating a FASTA file specifying the four data partitions (codon positions 1–3 and aggregated intergenic regions), to be consistent with our later Bayesian inference setup. In order to easily specify the partition information later on in BEAUti, we also create two FASTA files: one for the coding partition and another for the intergenic regions.

## Protocol

### Step 1: Assessing the Temporal Signal in the Data Using TempEst

Reliably estimating population size dynamics over time is dependent on having sufficiently strong temporal signal in the sequence data. In addition to assessing temporal signal, sequences that have accumulated significantly more or fewer than expected mutations given their sampling time, caused by issues such as laboratory contamination or mislabeling, need to be identified. Therefore a preliminary analysis in TempEst, an exploratory graphical application for examining temporal signal in time-stamped sequences (Rambaut et al. 2016), to identify these outliers should be routine procedure before committing to a potentially time-consuming analysis.

As its input, TempEst requires a ‘nonclock’ phylogenetic tree (i.e. with branch lengths scaled as genetic distances rather than temporal distances), which can be estimated using neighbor-joining, ML or Bayesian inference (Rambaut et al. 2016). This tree can be obtained using a variety of freely available software packages, and we here use IQ-TREE (Nguyen et al. 2015) to build an ML tree using our previously constructed FASTA file. We here specify partitions according to codon position for the coding region of our data set in order to allow different evolutionary rates across codon positions (Chernomor et al. 2016), as specified in the previous section. For each partition, we assume an HKY substitution model (Hasegawa et al. 1985) and accommodate among-site rate heterogeneity (Yang 1994, 1996). This estimation took ∼20 min to run on a standard computer.

**Fig. 2.**
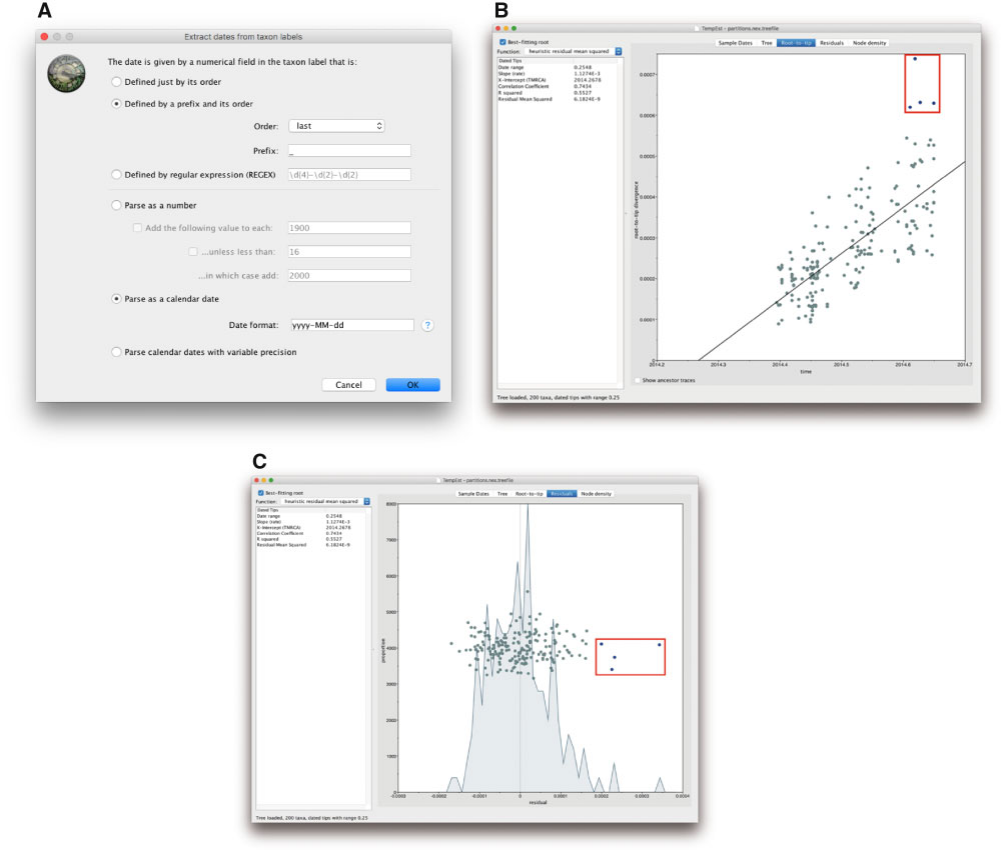
Using TempEst to determine whether our data set has sufficiently strong temporal signal and to identify outliers. (*a*) Dialog box showing how to extract sampling times from the sequence labels, (*b*) root-to-tip plot showing regression of genetic distance against time, and (*c*) residuals plot. In (*b*) and (*c*), four outliers can be identified and are indicated by the red box (for illustration purposes only, i.e. not a feature of TempEst).

Starting the TempEST application will open a file chooser to select a ‘nonclock’ phylogenetic tree. Select the .**treefile** generated by IQ-TREE—which contains the ML tree in Newick format—and click **Open**. Once the tree has been loaded into TempEst, click on the **Sample Dates** tab at the top of the screen. Click on the **Parse Dates** button to bring up a window (fig. 2*a*) that allows to provide a sampling time for each sequence in the alignment.

It is considered good practice to have the sequence labels contain the sampling times, which enables TempEst to extract these sampling times from the sequence labels after providing the date format. Note that IQ-TREE has separated the different fields in the sequence labels using an underscore symbol ‘_’ in the .**treefile**. To parse the dates in the sequence labels, we select **Defined by a prefix and its order**, select **last** from the **Order** drop-down menu and input ‘_’ (without the quotes) in the box by **Prefix**. This informs TempEst that the date is the last element in the label, preceded by an underscore. Select **Parse as a calendar date** and ensure that the **Date format** box is set correctly to **yyyy–MM–dd** (fig. 2*a*).

The tree is visualized in the **Tree** panel of TempEst, but of primary interest are the **Root-to-tip** (fig. 2*b*) and the **Residuals** panels (fig. 2*c*). In the **Root-to-tip** panel, we see the plot of a regression analysis of genetic divergence from the root of the tree against time of sampling, with each dot representing a time-stamped sequence. Initially, the tree is rooted arbitrarily, so we select the **Best-fitting root** button in the top left to select a root which minimizes the mean of the squares of the residuals. A linear trend with small residual variance indicates that evolution will be adequately represented by a strict molecular clock, and the same trend with greater scatter from the regression line suggests a relaxed molecular clock model may be most appropriate (Rambaut et al. 2016). Additionally, an objective but informal measure of the temporal signal is given by the correlation coefficient *R*^2^, but this should not be used to test the statistical significance of the regression. We conclude here that the Ebola virus phylogeny exhibits a moderate association between genetic distances and sampling dates ($R^{2}=0.55$) and is hence suitable for phylogenetic molecular clock analysis in BEAST (Suchard et al. 2018).

The slope of the regression line provides an estimate of the rate of evolution in substitutions per site per year, and the intercept with the time-axis constitutes an estimate of the age of the root. In this case, the rate estimate amounts to $1.12\times 10^{-3}$ substitutions per site per year and the origin is approximately March 2014, which both match previous estimates for Ebola virus in Sierra Leone (Dudas and Rambaut 2014; Dudas et al. 2017).

Importantly, both the plot in the **Root-to-tip** panel and the plot in the **Residuals** tab (fig. 2*c*) allow us to identify four sequences whose sampling date is incongruent with their genetic divergence (see also supplementary figs. S14 and S15, Supplementary Material online). These points can be manually selected, after which the corresponding sequences will be highlighted in every TempEst panel. Return to the **Tree** tab to see the labels of the problematic sequences and their position in the tree (supplementary fig. S3, Supplementary Material online). The four selected sequences in our data set—with accession numbers KR105291, MH607891, KR105296, and KR105286—all lie above the regression line, and therefore are more genetically divergent than we would have expected based on their sampling times. This may be due to a number of reasons, such as errors in the sequence assembly or an alignment error in part of the sequence (see Rambaut et al. 2016 for a more in-depth explanation). For example, upon inspecting our multiple sequence alignment, we notice that KR105296 has a large amount of missing data, which may result in the long branch leading up to it in the tree. Regardless of the underlying explanation, it is common practice to exclude such sequences from the multiple sequence alignment for the remainder of the analysis. For further discussion of these four sequences and why we remove them, see Supplementary Material online.

### Step 2: Using BEAUti to Set up the BEAST Analysis

**Fig. 3.**
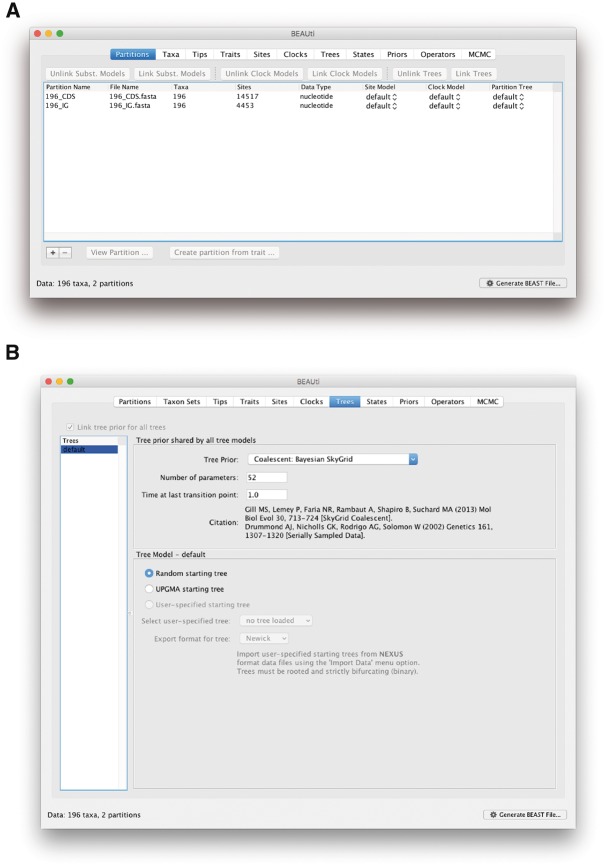
Setting up the Skygrid coalescent model in BEAUti. (*a*) Shows the data partitions we have imported using two different FASTA files for coding and intergenic regions. (*b*) Shows the “Trees” panel for setting up a Skygrid coalescent model to infer past population dynamics.

Now that we have established that there is a strong enough temporal signal to perform a BEAST analysis and the problematic sequence data have been removed, we can import our two FASTA files (containing the coding regions and the intergenic regions, respectively) into BEAUti, a graphical user interface (GUI) designed for creating XML files for input into BEAST (Suchard et al. 2018). Start the BEAUti application and drag-and-drop each FASTA file onto the **Partitions** panel. Alternatively, one FASTA file at a time can be imported using **Import Data…** from the **File** menu. You will see two entries show up in the **Partitions** panel, corresponding to the coding and intergenic regions (fig. 3*a*). Select both data partitions and tick the **Unlink Subst. Models** box, indicating that we will provide different substitution model choices for each data partition. Note that, we only discuss those panels in BEAUti in which settings have to be modified for the purpose of this protocol.

We now move on to the **Tips** panel, which is very similar in functionality to the **Parse Dates** panel in TempEst. We therefore obtain the sampling times of the sequences in the same manner by selecting **Parse Dates** and ensuring that **last** is selected and the prefix is set to ‘|’. First, select **Use tip dates** in the top left hand corner of the window. Then, click on the **Parse Dates** button just underneath this tickbox. Select **Parse as a calendar date** and ensure the correct format is entered, i.e. **yyyy–MM–dd**. It can be useful at this point to keep track of the youngest sampling date, which can easily be determined by sorting the table with the tip dates by clicking the **Date** column header.

The **Sites** panel allows selecting a nucleotide substitution model for each data partition. Notice that the data partitions can be selected in the left-hand panel, as we have previously unlinked their substitution models. First, select the coding (“196_CDS”) partition and choose ‘HKY’ from the first drop-down menu. Leave **Base frequencies** unchanged, select ‘Gamma’ from the **Site Heterogeneity Model** drop-down menu, and keep the default of four gamma categories. Finally, select ‘3 partitions: positions 1, 2, 3’ from the **Partition into codon positions** drop-down menu, and ensure that all three check boxes are ticked. This will allow different HKY models with among-site rate heterogeneity to be estimated for each codon position in the coding region of the alignment. Click on the ‘intergenic’ partition in the left-hand panel, and select all the same options, except for the **Partition into codon positions** which should be left as ‘Off’, as there are no codons in the intergenic regions. This selection of nucleotide substitution models is a popular option and corresponds to a fairly standard choice for analyzing Ebola virus sequences (Dudas et al. 2017).

Next, proceed to the **Clocks** panel and select the ‘Uncorrelated relaxed clock’ from the **Clock Type** dropdown menu. This allows each branch of the tree to have its own unique evolutionary rate, independent of the rate of its neighboring branches (Drummond et al. 2006). The **Relaxed Distribution** drop-down menu allows you to specify which probability distribution to draw these rates from. A lognormal distribution provides a proper trade-off between performance and complexity, and so we stick with this default option here.

In the **Trees** tab, click on the **Tree Prior** drop-down menu and select ‘Coalescent: Bayesian SkyGrid’ (fig. 3*b*). In the **Time at last transition point** section, we put **1.0** to signify that we wish to bound the estimation of the population size dynamics to at most 1 year before the most recently sampled sequence, which is a sensible choice given what we know by now concerning the 2013–2016 West African Ebola virus epidemic. A general guideline for this cutoff value is that it should be sufficiently greater than the anticipated root height of the tree, in order to capture as much information about the population dynamics as the data allow. If no prior information is available to set this value, a preliminary analysis can be performed to determine the estimated root height in order to set this value properly (Gill et al. 2013) (see Supplementary Material online). The **Number of parameters** option defines how often you allow the effective population size to change over the course of the time frame we have just imposed. We here estimate 52 population sizes across our 1-year interval, allowing a different population size to be estimated for every week. Finally, we keep the default option of a **Random starting tree** to start the inference process.

As the last step in BEAUti, select the **MCMC** panel which allows the specification of the computational settings and the output files that will be generated when running BEAST. In the **Length of chain** field, we need to put a number that is large enough to ensure we obtain a good effective sample size (ESS; the number of effectively independent draws from the posterior distribution that the Markov chain is equivalent to) for each parameter of interest. As there are a lot of parameters to be estimated, we will run this analysis for 100 million iterations.

In general, it is worth starting with an analysis of—for example—10 million iterations and then examining the output to have some idea of how long the actual analysis should run for (see Tracer section). This allows you to gauge how many iterations the analysis will require, and to assess how long it should run in real time which will help in securing appropriate computational resources.

In order to make a proper summary of the output of the BEAST analysis, we suggest aiming for 10,000 posterior samples in your final output file, for all parameters (including the trees) that will be sampled. Hence, in order to decide the appropriate value for the **Log parameters every** box, divide the MCMC chain length by 10,000 which in our case means logging every 10,000 states. Finally, the **File name stem** entry determines the file names of the various output files that will be generated and adjusting this file name stem then automatically modifies the **Log file name**, **Trees file name,** and **Operator analysis file name** text fields.

Once all these required settings have been specified, click on **Generate BEAST file** in the bottom right-hand corner to save the XML file containing all the provided information. This will generate a screen reminding you to review any priors which you have not changed, but as the default priors are proper and uninformative we can proceed by clicking **Continue**. Choose where to save your new XML file and click **Save**. This file is now ready to use as input for BEAST.

### Step 3: Performing the Analysis in BEAST

BEAST (Suchard et al. 2018) is a cross-platform program for Bayesian analysis of molecular sequences using MCMC and will perform the required estimation to ultimately determine the population size dynamics over time.

Start BEAST to bring up its GUI. In order to run analyses as efficiently as possible, BEAST requires the use of BEAGLE, a high-performance library which exploits multicore processors such as those in graphics processing units and standard (multicore) server processors found in all modern computers (Ayres et al. 2019). BEAGLE must be installed separately to BEAST, and is available from the BEAST website (http://beast.community/; last accessed July 29, 2019). To identify which resources your computer has available, select the **Show list of available BEAGLE resources and Quit** button in the BEAST GUI (see Supplementary Material online for further information). The default option for most machines will be to use the CPU, while other machines come equipped with one or more graphics processing units which are often more efficient at performing the core calculations required in phylogenetic inference. Instructions on how to optimize BEAST analyses are beyond the scope of this article, and further instructions may be found on the BEAST website (https://beast.community/performance; last accessed July 29, 2019).

After identifying the appropriate resource to run the BEAST analysis on your computer, restart BEAST and select the XML file that was generated using BEAUti. Click **Run** to start the analysis. After listing useful citations for the various models specified for the BEAST analysis, estimates for certain model components will appear, as well as (after 10,000 iterations) an estimate of the computational performance of the analysis, allowing you to gauge how long the analysis will take to finish. As an indication, this analysis took ∼16 h to complete on a high-performance CPU.

To ensure convergence to the same posterior distribution, we advise running at least two independent replicates by providing different starting seeds in the BEAST GUI (see Supplementary Material online for further discussion). For the purpose of this protocol, we ran the XML twice with different starting seeds.

After the BEAST analysis has finished, a summary table of the operators is shown on screen with information such as the attained acceptance probability of each operator. This information has also been written to the .**ops** output file, to go with the .**log** and .**trees** files that contain estimates for all the parameters and the sampled (time-stamped) phylogenetic trees, respectively.

### Step 4: Assessing Convergence and Mixing Using Tracer

**Fig. 4.**
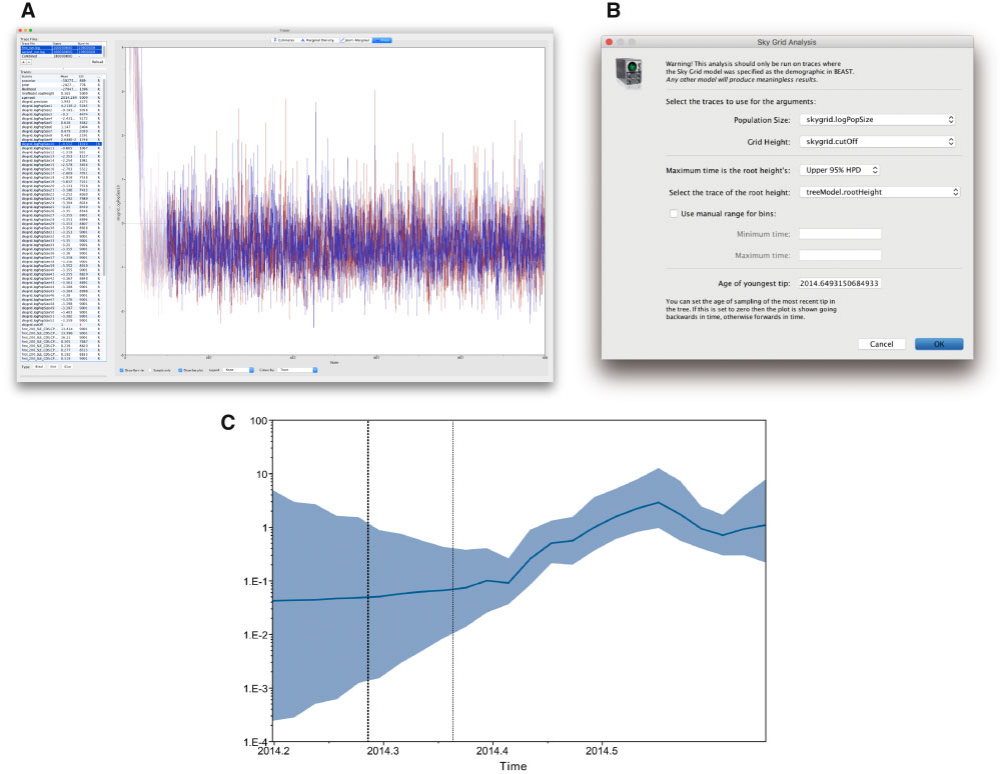
(*a*) The Tracer panel on the left shows the parameters logged during the run. Note that both runs are selected and as such, the panel on the right shows both traces in different colors. (*b*) Shows the options for the Skygrid reconstruction based on this analysis. (*c*) Shows past population dynamics visualized using the Skygrid model. The shaded portion is the 95% Bayesian credibility interval (obtained by clicking the “Solid interval” checkbox in the lower left-hand corner of the visualization window), and the solid line is the posterior median. The vertical lines represent the best estimate for the time of the root of the tree, and the upper highest posterior density, respectively.

Now that our two independent BEAST analyses have run to completion, we will use Tracer (Rambaut et al. 2018) to examine their output. Tracer is a graphical tool to visualize and diagnose issues in the output files generated during an MCMC analysis, and can be used with most Bayesian phylogenetics software packages. To begin, launch Tracer, click on **Open** in the menu bar, and select the .**log** files from your BEAST runs. Alternatively, drag and drop one or more .**log** files into the Tracer window (outcome shown in fig. 4*a*).

In Tracer, the panel on the left is split up into two subpanels, where the upper-left panel contains the names of the files that have been loaded along with the length of the analysis in number of iterations and an initial burn-in of 10%. The burn-in percentage can be manually adjusted here so that a portion of the parameter and density samples—corresponding to that part of the Markov chain that has not converged yet to the stationary distribution—is not taken into account when computing the summary statistics (see Rambaut et al. 2018 for more details). This percentage is important when one is interested in constructing a maximum clade credibility tree from the .**trees** file, which is beyond the scope of this protocol.

In figure 4*a*, we show the **Trace** panel containing the two traces of a single (log) population size parameter of the Skygrid coalescent model, showing that our two independent replicates (shown in red and blue) have converged to the same posterior values, and that for this parameter a default 10% burn-in is sufficient. We note here that all parameters and densities of interest should be examined for their convergence to the same posterior distribution as well as properly inspected to determine an appropriate overall burn-in value. The lower-left panel contains the names of all the parameters and densities that the log file contains, along with their mean values and their associated ESS values. If multiple files corresponding to independent analyses from the same XML file have been loaded into Tracer, a combined trace will appear below the loaded files, effectively aggregating the samples from those files for each density and parameter. In effect, this also results in all statistics—such as the mean and ESS value—being computed on the aggregated sample collection.

As we are interested in reconstructing past population dynamics, we focus on ESS values for all parameters of the Skygrid coalescent model, as well as statistics related to important aspects of tree estimation, such as the root height. The ESS of a parameter equals the chain length (without burn-in) divided by the autocorrelation time, i.e. the number of states in the chain that must separate two samples in order for them to no longer be correlated. Simply put, the larger the reported ESS value the better, but in practice a minimum value of 200 for all parameters of interest is used to determine if an analysis has run for long enough. Tracer flags up any ESS values <100 in red and <200 in yellow. Low ESS values are usually the result of an insufficient number of iterations in the analysis and are most easily increased by running the analysis for a larger number of iterations (see https://beast.community/ess_tutorial; last accessed July 29, 2019 for more information). Note that the cutoff value of the Skygrid model is a constant value and hence its ESS value is of no importance.

The ESS values will correspond to the behavior you see in the **Trace** panel, where line plots connect the sequential samples of one or more selected parameters against iteration number. These trace plots are typically used to assess convergence, determine a corresponding burn-in, and to assess proper mixing. Ideally they should look similar to the ones in figure 4*a*,i.e. a reasonably short burn-in sequence followed by a stable trace that does not show any trends. In Supplementary Material online, we provide examples of analyses that can be identified using Tracer as being problematic.

Now that we have assessed that our two independent replicates have converged to the same posterior distribution for all parameters and densities, we can exploit having two collections of samples at our disposal. We note that this is an optional step in this particular protocol, as the ESS values for all parameters of interest are sufficiently high for each individual replicate. The BEAST package contains the LogCombiner application, which allows aggregation of the .**log** and .**trees** output files from independent BEAST replicates. Aggregating these files will increase the number of independent samples from the posterior distribution.

Start the LogCombiner application and a dialog box will appear. In the **File Type** menu you can specify whether you are combining .**log** or .**trees** files. For the purpose of this protocol, we will be combining the .**log** files of our two independent replicates and use the resulting file to visualize the population size dynamics over time. Hence, in the **File type** dropdown menu, select **Log files** and add the two .**log** files using the **‘+’** button. For each .**log** file, we manually set the burn-in to 10% of the number of iterations performed, i.e. 10 million. As a final step, specify the name of the output file that needs to be created by entering it manually after clicking **Choose File…** and click **Run** to generate the combined file.

### Step 5: Visualizing the Skygrid with Tracer

Tracer also allows the visualization of demographic reconstructions of various coalescent models. To perform these reconstructions, it is important to combine each analysis performed with the appropriate demographic reconstruction option in Tracer. From the **Analysis** menu, we here select the **Skygrid Reconstruction** option.

By default, the **Skygrid Analysis** dialog box only requires the **Age of youngest tip** to be provided in order to provide a visualization corresponding to real time (fig. 4*b*), as all the other fields are inferred automatically from the log file. The **Age of youngest tip** corresponds to the most recently sampled tip, 2014.65 for our data set, which corresponds to the 26th of August 2014 in decimal format. If this value is left as 0, the Skygrid visualization will be shown going backwards in time.

The Skygrid plot will pop-up almost immediately (fig. 4*c*), with the solid (blue) line the posterior median of the population size over time, and the upper and lower lines representing the corresponding 95% highest posterior density (HPD) interval. For additional clarity, check the box **Solid interval** to convert this to a shaded area. The vertical dotted line to the left of the 2014.3 mark is the mean estimate of the root age, and the two dotted lines to the left (overlapping with the Y axis here) and right correspond to its 95% HPD interval. In general, as we go back in time, we see an increased uncertainty in the estimated population sizes, as well as a flattening of the overall curve, as fewer data are available to inform this part of the model. From this visualization, we conclude that the effective population size was fairly static until the end of May 2014 (2014.4), after which it increased rapidly until forming a fairly stable plateau with minor fluctuations. In the Supplementary Material online, we relate these population size dynamics over time to reported case counts.

## Concluding Remarks

In this protocol article, we have presented a complete workflow for estimating past population dynamics using BEAST and its accompanying applications. To this end, we have estimated the Skygrid coalescent model in combination with an uncorrelated relaxed clock model (Drummond et al. 2006), a popular model combination for data exploration. Although the relaxed clock model is able to accommodate many different scenarios of rate variation across the branches of the tree, alternative clock models—such as the random local clock (Drummond and Suchard 2010) and host-specific local clock (Worobey et al. 2014) models—may be better suited for your own data.

Coalescent models have come a long way in relaxing their initial assumptions and, as shown in this protocol, allow the accommodation of variable population size and serially sampled data to generate genealogies arising from a forward-time population model (such as the Wright-Fisher model). However, we caution against overinterpreting the outcome of such a reconstruction, as even flexible nonparametric coalescent models such as the Skygrid are subject to a number of assumptions, which will have their impact on the estimated effective population size of the virus (see Gill et al. 2013 for more information).

Additionally, if the true underlying model would be a simple parametric model—such as a constant population size model—then the Skygrid model will be overparameterized and yield large 95% HPD intervals. In those cases, switching to simpler models will be advantageous to reduce the uncertainty of the reconstructed population size(s) and allow for more easily interpretable results, better convergence, and mixing which in turn lead to reduced computation times.

We hope that this protocol provides a useful set of practices and guidelines to become familiar with BEAST and its accompanying applications. Importantly, most of the concepts and guidelines discussed here are not exclusive to BEAST, and other Bayesian inference programs can perform similar analyses, for example RevBayes (Huelsenbeck et al. 2016), MrBayes (Ronquist et al. 2012), and BEAST 2.5 (Bouckaert et al. 2014).

## Supplementary Material

Supplementary data are available at *Molecular Biology and Evolution* online.

## Supplementary Material

msz172_Supplementary_Data
