# Supplementary material for: Bayesian Estimation of Past Population Dynamics in BEAST 1.10 Using the Skygrid Coalescent Model
Source: Mol Biol Evol. 2019 Jul 31;36(11):2620–8. doi: 10.1093/molbev/msz172 (PMC6805224; doi:10.1093/molbev/msz172)
Supplement: msz172_Supplementary_Data [file msz172_supplementary_data.zip › Supplementary_information.pdf]

Bayesian estimation of past population dynamics in BEAST 1.10 using the Skygrid coalescent model: Supplementary Materials

Verity Hill, Guy Baele

July 15, 2019

Contents

1 General information **1**

2 Protocol workflow **2**

3 Manual data processing **3**

4 Date format in TempEst and BEAUti **4**

5 Assessing the temporal signal in the data using TempEst **4**

6 Initial root height estimation using TreeTime **6**

7 Running BEAST **7**

8 Trace diagnostics **13**

9 Skygrid reconstruction and interpretation **16**

10 Ancient DNA analyses **17**

11 Main manuscript figures **19**

1 General information

In this Supplementary Materials document, we provide additional information and figures complementing the main manuscript. In section 2, we provide a schematic overview of the workflow that is described in detail in the main text. In section 3, we provide additional information on constructing, aligning and manually processing the alignment. We go on to discuss potential pitfalls concerning the date format used in both BEAUti and TempEst in section 4 and show how to set the dates correctly in order to assess the temporal signal in the data set in section 5. In section 6, we discuss how to find an initial estimate of the age of the root using the Ebola virus dataset from the main text. In section 7, we focus on running BEAST (Suchard et al., 2018) through its graphical user interface (GUI) and the settings that may be used when doing so. We discuss the use of BEAGLE high-performance computational library (Ayres et al., 2019), as recent versions of BEAST (i.e. 1.10 and later) explicitly require its use in order to run, although in practice this was also a requirement in earlier versions to successfully perform many computationally demanding analyses. We also show how to identify and select a hardware

device of choice for your analysis, by using a built-in feature within BEAST. In section 8, we show a few examples where the analysis – or more specifically performing multiple independent replicates of the same analysis – did not (yet) produce high-quality results. We focus on how to identify these issues and possible approaches to remedy the situation. In section 9, we provide some more details on the Skygrid reconstruction shown in the main manuscript and discuss how the estimated population dynamics over time can and should be related to observed results (if available). To this end, we superimpose case counts from the World Health Organisation (WHO) onto our estimates and discuss the similarities and differences. In section 10, we elaborate on the use of the proposed workflow for analysing ancient DNA data sets which, under the right conditions, also constitute measurably evolving populations (MEPs), a term that is usually associated with fast-evolving RNA viruses. Finally, we conclude with showing larger versions of the figures in the main manuscript in section 11, for additional clarity.

2 Protocol workflow

This section provides a schematic overview of the workflow in the main text. Starting with a cleaned multiple sequence alignment (see section 3), the first step consists of estimating an unrooted phylogenetic tree using software such as IQ-TREE (Nguyen et al., 2014). We then use this tree to check the molecular clock assumptions using TempEst (Rambaut et al., 2016). We remove outliers of the regression analysis of time against genetic distance, as these may be indicative of biological or technical issues when generating the data. We go on to specify the models in BEAUti which we will use to perform inference in BEAST, and run a number of replicates of the analysis. Afterwards, we check that the model parameters converged and exhibited proper mixing, and visualise the past population dynamics over time using Tracer (Rambaut et al., 2018).

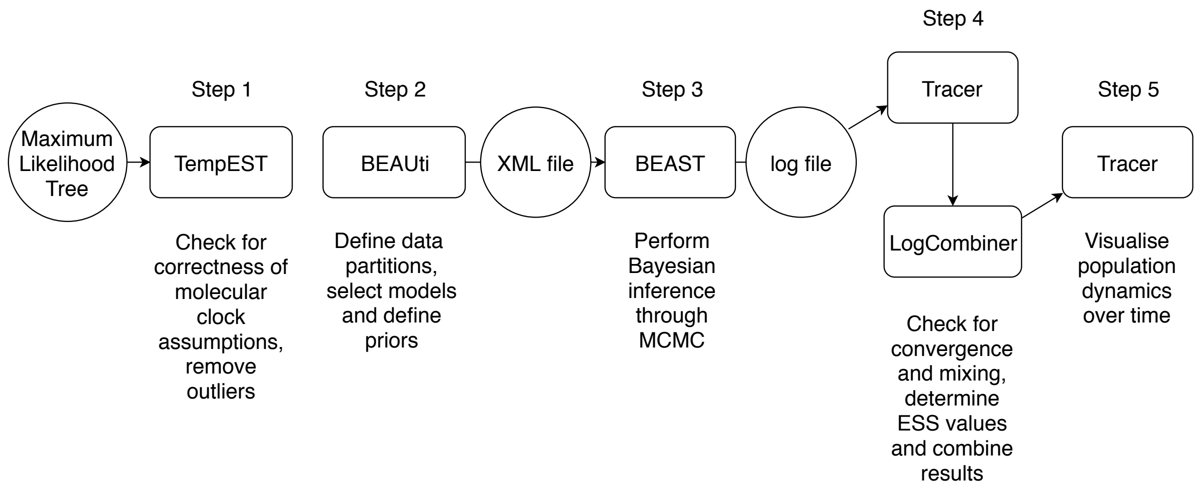

Figure S1: Workflow of the various steps and software packages (in boxes, with input files shown in circles) required to infer past population dynamics using the non-parametric Skygrid coalescent model (Gill et al., 2013). An important first step is a preliminary check of the molecular clock assumptions using TempEst, which takes an unrooted phylogenetic tree as its input. Here, we opt for a maximum-likelihood approach to construct this initial phylogeny. After removing outliers identified by TempEst, we use BEAUti to construct the input XML file for BEAST and subsequently perform the analysis across multiple independent replicates. Upon completion, the results are inspected and compared in Tracer, which is also able to visualise the past population dynamics over time.

3 Manual data processing

This section provides some additional information on how we constructed, aligned and post-processed the alignment. We performed these edits in Geneious (`https://www.geneious.com`), but a number of freely available software packages can be used to perform these tasks, such as MEGA (Kumar et al., 2012) and AliView (Larsson, 2014). We first downloaded our 200 individual Ebolavirus sequences of interest from GenBank (`https://www.ncbi.nlm.nih.gov/genbank/`) and aligned these sequences using MAFFT (Nakamura et al., 2018), a command-line tool which can be run using the command:

```
mafft [unaligned sequences FASTA file] > [output file name]
```

First, it is helpful to locate the genes within the alignment by using the complete record – which includes annotations for the genes' locations – of any of the sequences from GenBank. Import this file into Geneious using drag-and-drop into the top panel, or by clicking "Import" in the **File** drop-down menu. Click on this file and your alignment in the top panel, right click and select "Multiple align". Leave all the options as they are and click "OK". This aligns the annotated sequence with the rest of the alignment, which will take a few minutes. Once this alignment step has been completed, you can see that the annotations from the GenBank file now appear in the main alignment.

Specific to our EBOV data set, we have added a question mark into every sequence to account for the ribosome slippage that we know occurs in the GP gene of EBOV. This sort of information can be found in GenBank entries for EBOV sequences; as an example, we provide the GenBank entry for accession number KR105247 in our online supplementary data repository (file: KR105247.gb): `https://github.com/GuyBaele/Skygrid-Protocol`. If we look at the GP CDS annotation which we have just included in the alignment, we can see a small grey box saying "GP Misc Feature". If you hover it, this tells us that additional adenosine residues can be inserted or deleted here during transcription by the polymerase. This might disrupt the reading frame, which would in turn interfere with our analysis which is partitioned by codon position. In this alignment, to maintain the reading frame (which we can test by dividing the length of the coding region by three), we only need to add one insertion. We do so at the start of the run of A's, following Dudas et al. (2017). In this alignment, this insertion occurs at position 6924. We went on to mask putative adenosine deaminases acting on RNA (i.e. ADAR)-edited sites. ADAR is a mammalian enzyme which non-randomly edits the RNA of viruses, producing multiple T-to-C mutations simultaneously close together in the genome. If not accounted for, ADAR editing can alter molecular clock estimates, as it induces additional variation. We follow Dudas et al. (2017) and treat instances of four or more T-to-C mutations within 300 base pairs of each other as a putative hypermutated tract. We therefore replace the C's with question marks to mask this, but (again, following Dudas et al. (2017)) we leave the first T-to-C mutation unmasked to provide some information on sequence relatedness. In this alignment, this occurs in the sequences with the accession numbers KR105247, beginning at position 5518, and KR105300, beginning at position 6681.

Finally, we manually moved intergenic regions towards the end of the alignment for ease of use when constructing a BEAST XML file later in the workflow. We will again turn to the annotations we have imported from GenBank. By visually inspecting the annotations, you can see where the intergenic regions fall. Highlight them in all of the sequences, and then cut and paste at the end of the alignment. Once you are done, make sure to delete the additional 'annotation' sequence from the alignment.

This completes the manual processing of the alignment in order to prepare the data for the analysis in BEAST, with the final alignment length amounting to 18970 bases. We have provided the unaligned sequences (200_unaligned.fasta), the aligned but uncleaned sequences (200_alignment_unclean.fasta) and the final alignment (200_final.fasta) in the online GitHub materials (`https://github.com/GuyBaele/Skygrid-Protocol`), as well as the GenBank file of one of the sequences for annotation purposes.

4 Date format in TempEst and BEAUti

When processing the sampling times for the sequences in a data set from the sequence names, a common date format is listed by default in both TempEst and BEAUti, which follows the format used in Java `https://docs.oracle.com/javase/7/docs/api/java/text/SimpleDateFormat.html`. By default, the format is set to **yyyy-MM-dd**, as can be seen in Figure S2. However, this can easily be adjusted – both in TempEst and BEAUti – by providing your own custom format. In doing so, we advise reading the documentation concerning the date format, which can be brought up by clicking the '?' next to the 'Date format' text field.

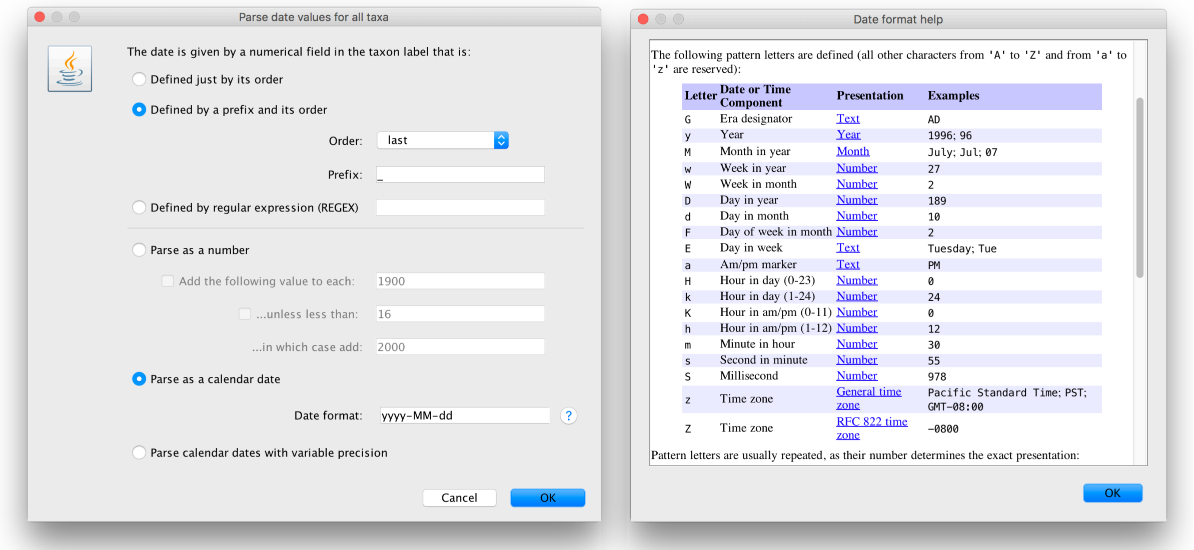

Figure S2: Parsing the dates in the sequence names in BEAUti and TempEst. Users have to be careful in terms of providing a correct 'Date format' but can look at the date and time format by clicking the question mark in the figure on the left. This will bring up the specification options (see figure on the right). Of importance is to note that 'm' represents minutes and 'M' represents months.

5 Assessing the temporal signal in the data using TempEst

This section provides further information on TempEST and using it to remove potentially problematic sequences (see main text). After loading the unrooted tree, setting the sampling times for the sequences into TempEst, and selecting the best-fitting root, we can inspect the Residuals, Root-to-tip and Tree panels to identify sequences whose genetic divergence and sampling date are incongruent. To identify such sequences, TempEst performs a regression of root-to-tip genetic distance against sampling time, which can be used as a simple diagnostic tool for molecular clock models. A linear trend with small residual variance indicates that evolution will be adequately represented by a strict molecular clock, and the same trend with greater scatter from the regression line suggests a relaxed molecular clock model may be most appropriate (Rambaut et al., 2016). TempEst is, as per the developers' instructions (Rambaut et al., 2016), an exploratory tool which allows the combination of different visualisations to determine if certain sequences should be removed from the data set due to either data quality problems, including errors in data annotation, sample contamination, sequence recombination, or alignment error. TempEst therefore identifies sequences that warrant a detailed inspection and may need to be removed as a result.

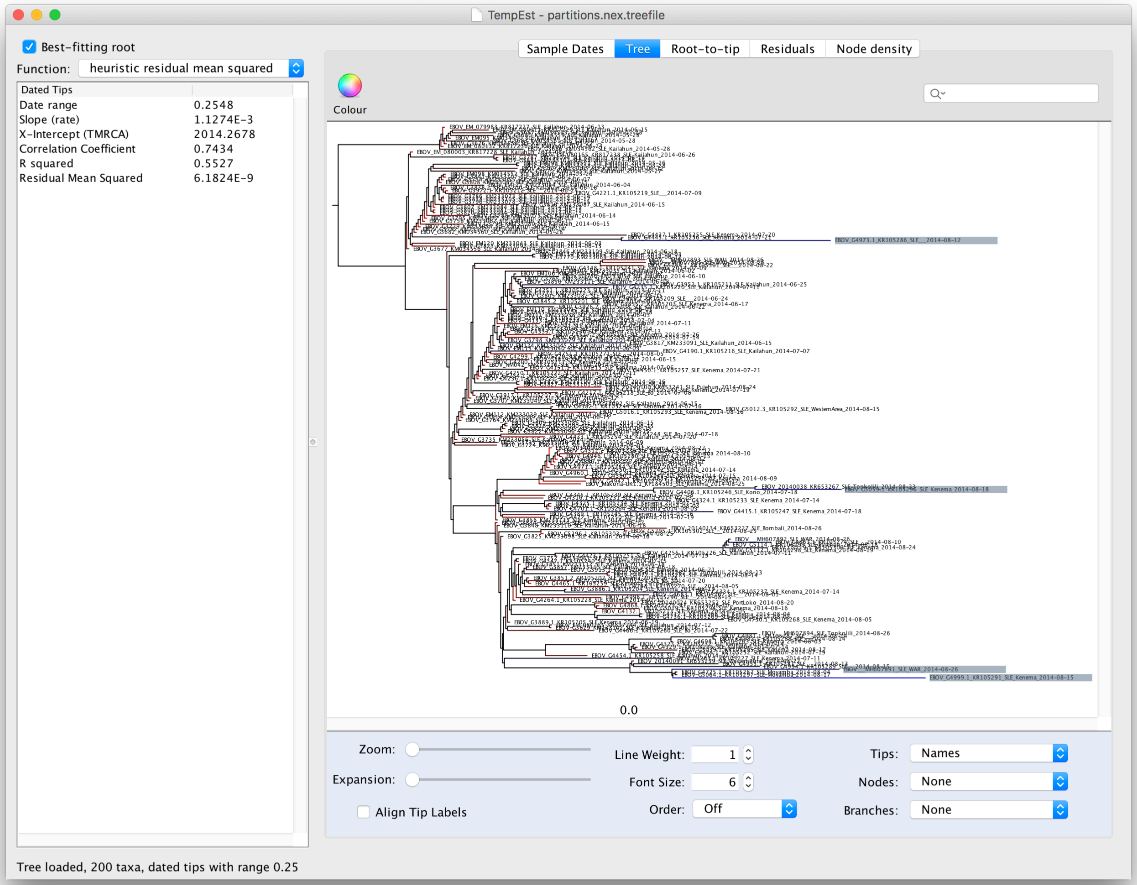

Figure S3: The Tree panel in TempEst when selecting the four outliers in the Residuals panel. The four sequences – highlighted in grey – corresponding to the four selected outliers clearly have extremely long branches compared to the rest of the tree.

In the main text, we have identified four such sequences in this manner. Note that these four sequences lie substantially above the regression line, which indicates that they have considerably more genetic divergence from the root than one would expect given their date of sampling. We go on to inspect the location in the phylogeny for those sequences by keeping those four data points selected in the 'Residuals' panel and navigating to the 'Trees' panel in TempEst. Figure S3 shows the full phylogeny with the taxon names corresponding to the four selected data points highlighted in grey. The four data points clearly correspond to extremely long branches when compared to the rest of the tree, and a detailed inspection of the multiple sequence alignment reveals that these four sequences each have multiple unique substitutions that are not shared with any of the other sequences, explaining the long branches and the fact that these sequences do not cluster together as a single clade. It is hence plausible that these substitutions correspond to sequencing errors and we subsequently remove these four sequences from the alignment.

6 Initial root height estimation using TreeTime

This section provides information on how to obtain an initial estimate of the root age (or root height) of a phylogeny when such information is not readily available as prior knowledge. In the main manuscript, we use a data set from a well-studied epidemic that has clear epidemiological evidence of spillover from an animal reservoir into the human population. It is therefore relatively easy to assign "Time at last transition point" for the Skygrid analysis. However this is often not the case, especially with pathogens that are endemic in humans, or those that have less well-defined outbreaks. We here provide a method to gauge the root prior to performing the BEAST analysis.

After producing a non-clock phylogenetic tree with IQ-TREE and removing any outliers after examination in TempEst (see main text and the previous section), we use TreeTime (Sagulenko et al., 2018) to convert our maximum-likelihood (ML) tree into a time-stamped phylogenetic tree. Note that several other packages are available to perform this task, such as least-squares dating (To et al., 2015) and treedater (Volz and Frost, 2017). Hence, we must first rerun IQ-TREE without the detected outliers to produce an ML tree for use with TreeTime, i.e. we rerun IQ-TREE with 196 sequences. To run TreeTime, we need three input files: the ML tree (i.e. the .treefile) obtained from the second IQ-TREE run, the alignment file of the 196 sequences (used for the IQ-TREE run), and a comma-separated values (CSV) file containing one column of taxon labels and another column with the corresponding sampling times. Note that TreeTime requires the titles of these columns to be "name" and "date". If there are sequences without exact date of sampling, add "XX" in place of month or day, as this will allow TreeTime to incorporate the sequence with date uncertainty, so for example "2014-03" will become "2014-03-XX".

However, we first need to make some changes to our alignment, so that the names of the sequences in all three files match up. To this end, we need to rename the sequences in the alignment file to replace the "—" symbol with "_"s, as IQ-TREE automatically performs these symbol substitutions in the ML tree file it produces. We also need to remove the "?"s from the sequence names, as Treetime will not accept names that contain question marks; we have replaced the question marks with "Unk" for "Unknown". These edits can both be performed using the "Find and replace" function in many text editors, but be careful as there are "?"s in the sequences which must remain there (e.g. at the GP slippage, or at ADAR-edited sites); alternatively, you can write a custom script to perform these changes.

TreeTime is freely available from `https://github.com/neherlab/treetime` and can be used via the command line, or as part of a python script. A webserver is also available to perform the analysis, at `https://treetime.biozentrum.unibas.ch/`. We used the command:

```
treetime --tree [IQTree_treefile] --aln [alignment_in_FASTA_format]
         --dates [CSV_dates_file] --gtr HKY
```

where the last argument specifies which nucleotide substitution model to use. We have here assumed an HKY model to maintain consistency across our entire workflow. The TreeTime analysis took less than one minute to run on a high-performance CPU. The most pertinent results of this analysis are shown in Figure S4. TreeTime will also produce a FASTA file of inferred ancestral sequences for each node. Looking first at the root-to-tip plot, we can see that the root age estimate is very similar to the regression analysis in TempEST, with a gradient of 1.17e-03 (substitutions per site per year) compared to 1.0207e-03 (substitutions per site per year). The intercept of the TreeTime plot is 2014.3, which is slightly more recent than the TempEST outcome of 2014.25. We can also see a time-stamped tree as a TreeTime output, which is shown in Figure S4.

We can therefore treat 2014.3 as an initial estimate of the root age of the tree, and use this information to inform our choice for the **Time at last transition point** in the Skygrid analysis (see main text) to 1 year before the most recent sampling time. As mentioned in the main text, a general guideline for this cutoff value is that it should be sufficiently greater than the anticipated root height of the tree, in order to capture as much information about the population dynamics as the data allow. We hence assume a conservative value for the **Time at last transition point** of 1.0, to take into account the possibility

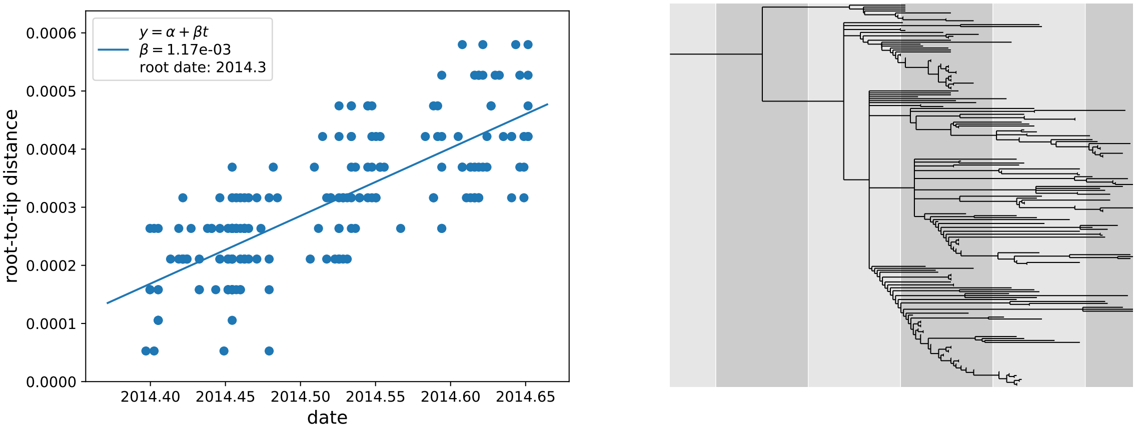

(a) Root-to-tip plot from TreeTime analysis

(b) Time-stamped tree from TreeTime analysis

Figure S4: Results of TreeTime analysis to find a reliable estimate of the age of the root of the tree. (a) shows the root-to-tip regression plot from TreeTime. This corroborates our results from the TempEst analysis, and provides us with an initial root age estimate for use in later analyses. (b) shows the time stamped tree produced by TreeTime.

of these heuristic approaches underestimating the true root height. We caution against assuming a too low value for this cutoff, as also the 95% highest posterior density for the root height will be required to accurately estimate and visualise the effective population size over time and the accompanying 95% uncertainty intervals.

7 Running BEAST

This section provides additional information on using BEAST through its graphical user interface (GUI), although most of the suggestions also apply when using the command-line interface to run BEAST. Figure S5 shows the BEAST GUI, which opens automatically upon double-clicking the BEAST icon or application. First, we will use the GUI to check which hardware resources are available for running BEAST with BEAGLE, so as to optimize performance. To do this, check the box beside **Show list of available BEAGLE resources and Quit**, leave all the other options as they are (there is no need to actually load an XML file) and click **Run**.

Figure S6 shows the output that this produced on one of our systems. Starting with the BEAST version (v1.10.4 in this case), the BEAST development credits are shown, followed by the version of the BEAGLE high-performance computational library currently installed (Ayres et al., 2019). On this system, the installed BEAGLE version is v3.1.0; if this information does not show or you don't see a similar output, this is usually a sign of BEAGLE not having been installed properly or that BEAST for some other reason can't locate the BEAGLE library. Before any BEAST analysis can be attempted, BEAGLE needs to be installed. When not running BEAST using the GUI, you can provide the location to the BEAGLE library (by default in the /usr/local/lib directory on Mac OS and Linux) as follows:

```
java -Djava.library.path=/usr/local/lib -jar beast.jar [analysis.xml]
```

The following section of the output shows the computational resources that BEAGLE is able to detect on the system. Regardless of the number of processor cores available, resource 0 will always identify the

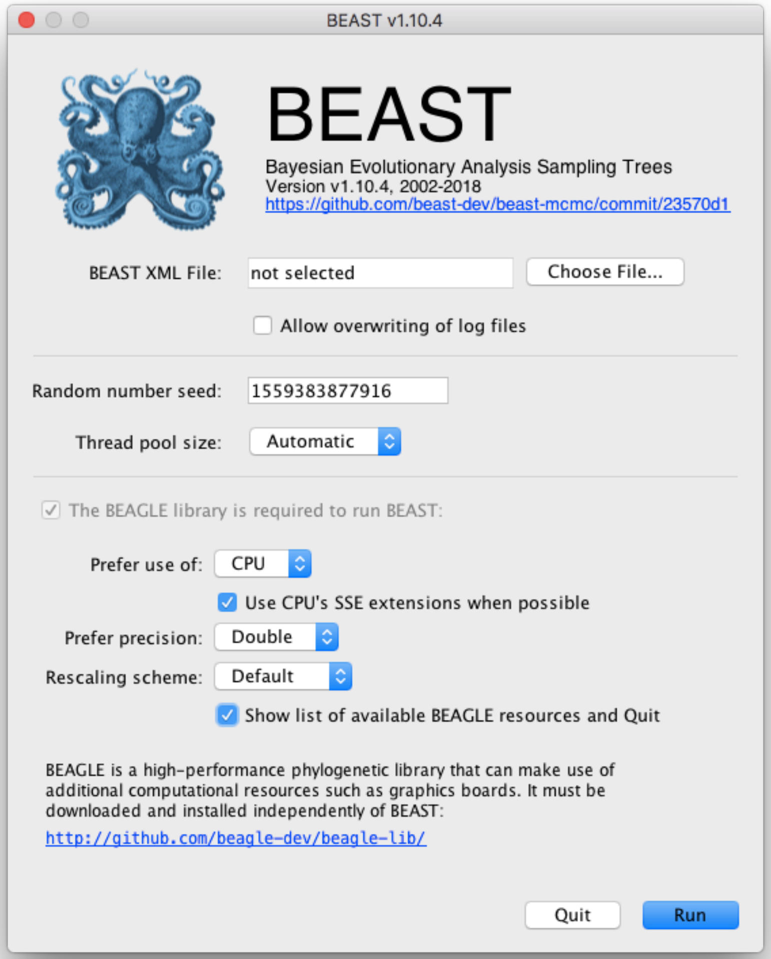

Figure S5: Graphical user interface for BEAST to show how to find out which BEAGLE resources are available on your machine. Note that the box saying **Show list of available BEAGLE resources and Quit** is checked. As you can see, there is no need to load a BEAST XML input file at this point.

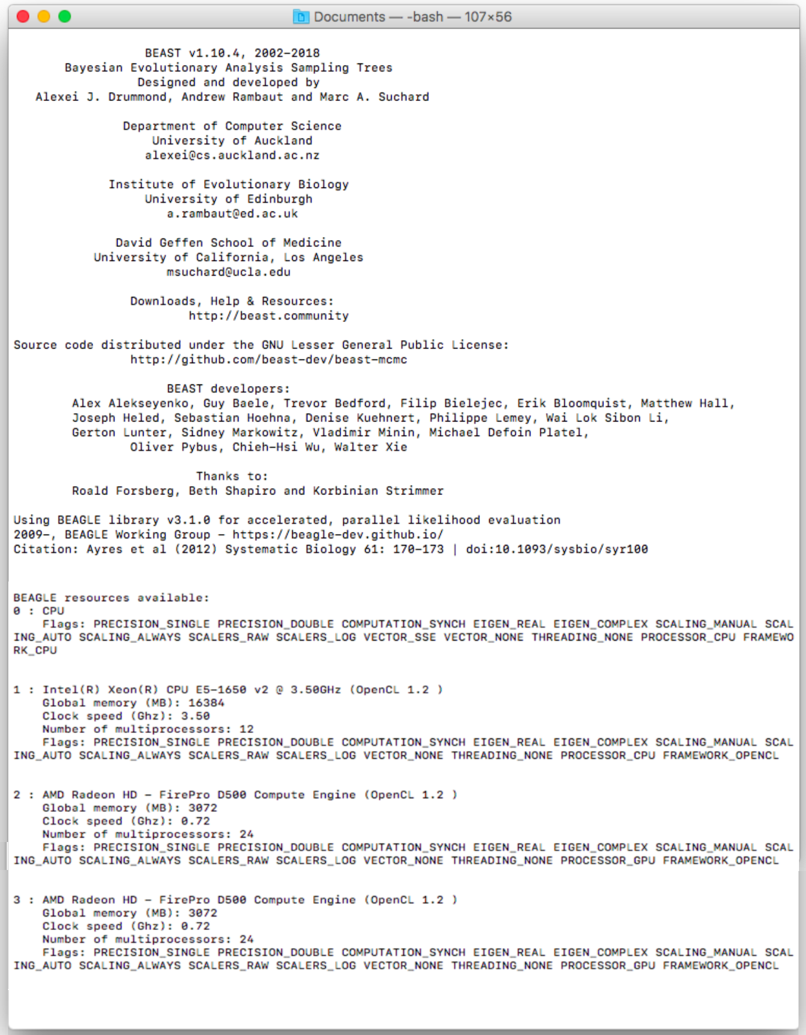

Figure S6: Output of clicking **Show list of available BEAGLE resources and Quit** in the BEAST GUI on one of our multi-core systems. The section describing which computational resources are available begins half way down under "BEAGLE resources available". This will vary based on hardware specifications of the actual machine, and our computer comes equipped with a CPU and two GPUs.

CPU and will hence always be available. Note that most hardware specifications listed will also mention how many multiprocessors are available, as BEAST can be run in parallel across multiple threads in order to increase performance. The other resources will depend on the hardware specifications of the computer or server you are using. Certain resources may be listed multiple times, depending on the support they have for certain computational frameworks/libraries. Two of the most popular frameworks are CUDA (proprietary to NVIDIA) and OpenCL (an open standard for parallel computing devices), and both are supported in BEAGLE (we refer to Ayres et al. (2019) for more information).

In our case for example, resource 1 is the OpenCL version of resource 0 (the CPU), but both resources actually point to the same underlying hardware. Resources 2 and 3 shown in Figure S6 are GPUs: two AMD FirePro D500 GPUs with 3 Gb of on-board memory, which are both accessible through OpenCL. In this case, NVIDIA's CUDA platform is not available for these GPUs so they are only listed once. If this platform is available (i.e. for GPUs from NVIDIA), each GPU will be listed twice in this list. We note that in our experience AMD's OpenCL platform is less mature than the CUDA platform for use in phylogenetics, which may lead to inconsistent performance, but see Ayres et al. (2019) for more information. As a result, we will here opt to use resource 0 over resource 1 (and not use the OpenCL framework). Now that we have identified the hardware resource on which to run the BEAST analysis, we can close this window.

We now move forward with our analysis by restarting BEAST and locating our XML file by clicking on the **Choose File** button. If you have already completed a test run (for example to ascertain how long the analysis needs to run for to obtain sufficient effective sample sizes - see main text) or performed a previous BEAST analysis that generated output files with the same file names, then you will need to click **Allow overwriting of log files**. If this box is not checked and you have files of the same name in the same file from the test run, BEAST will throw an error and exit. If you do not wish to overwrite existing files, you will need to edit the XML either manually or in BEAUti to change the output file names. The **Random number seed** box determines where the MCMC chain will start in parameter space, which will change by default each time you launch BEAST, leading to certain starting values being randomly generated (typically the case for the starting tree topology for example). If you'd like to run the analysis again to get the exact same output, for example in case you forgot to write certain output to file or want to run the exact same analysis for longer, you can manually set this value.

The **Thread pool size** block is for optimising performance on a multi-core CPU. In the BEAGLE resources output in Figure S6, we could observe the number of processor cores available in the CPU. This drop-down menu allows you to specify how many threads to run the analysis on. It is best to leave this on the **Automatic** default, as it does not follow that using more threads always leads to a faster run. In the next drop-down menu, you can specify whether you want BEAST to run on a GPU or a CPU. Not all machines will have a GPU, but you can check the available BEAGLE resources as specified above.

A BEAST window that is ready to run is shown in Figure S7. When the BEAST run begins, it may throw errors if, for example, the XML file was not properly written to file or if BEAGLE is not installed or can not be located by BEAST. For information on possible errors and how to solve them, see `http://beast.community/errors` for more information. We have used the following starting seeds to run the two BEAST analyses for which we provide the output in the online GitHub repository: '123' and '456'. Using these starting seeds, we run the BEAST analyses on CPU (i.e. resource 0) using the following commands:

```
> java -Djava.library.path=/usr/local/lib -jar beast.jar -seed 123 -beagle_cpu
      -beagle_double -beagle_order 0 skygrid_52_timepoints.xml
> java -Djava.library.path=/usr/local/lib -jar beast.jar -seed 456 -beagle_cpu
      -beagle_double -beagle_order 0 skygrid_52_timepoints.xml
```

Running two independent replicates of the same Bayesian inference through MCMC is often considered a minimum requirement to check for convergence towards the same parameter values and resulting densities, and it's generally advised to perform several independent replicates. However, it is likely you

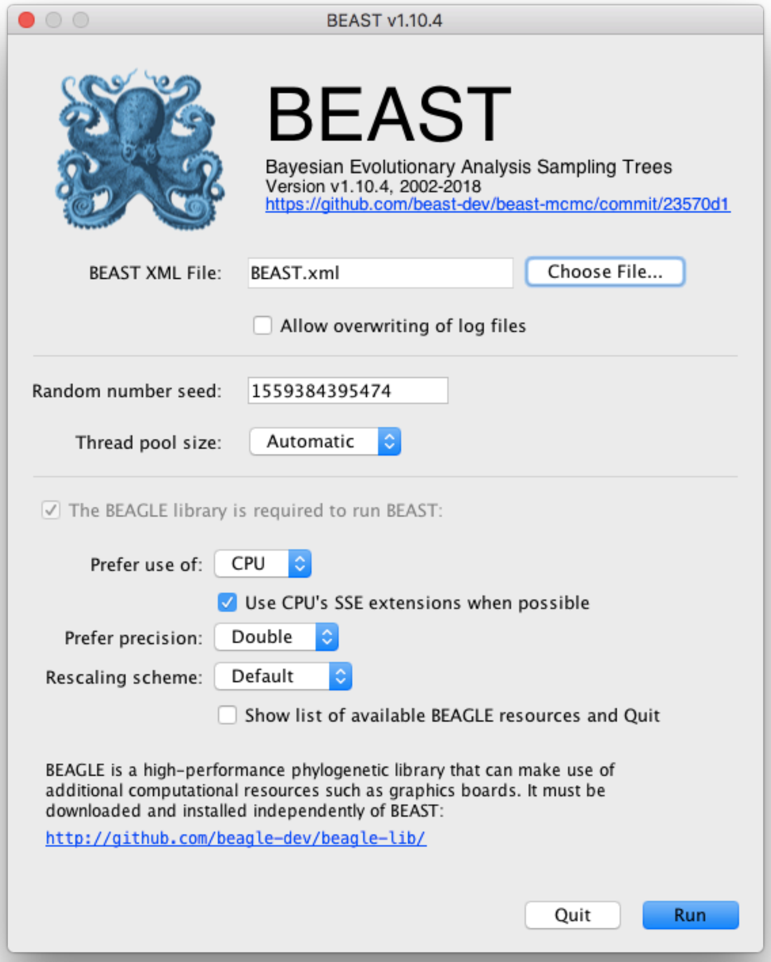

Figure S7: BEAST Graphical User Interface (GUI). With the XML file loaded and the computational resource selected, we're now ready to run the BEAST analysis.

will need to take into account that a finite amount of resources are available within your research group, and find a good balance between the number of replicates to run and how long to run each replicate. While a larger number of independent BEAST replicates can be run, you need to ensure that each replicate is able to run for an extended amount of iterations in order to ensure proper convergence to the joint density (i.e. the product of all priors and likelihoods), and hence use the available hardware resources accordingly. Running chains well past burn-in remains critically important for high-dimensional problems and even more so when confronted with large data sets. There stands ample evidence in the literature of MCMC analyses that appear to be converging until a new part of the state space is discovered, even after those analyses had already run for a long time. Running multiple shorter chains does not allow to detect such issues due to the limited run length of each individual analysis, so again we advise prioritising the running of long chains. Explicit discussion of MCMC burn-in issues and why many small MCMC chains are inferior to a single long chain is beyond the scope of our protocol. We therefore refer interested readers to, for example, Charles Geyer's website `http://users.stat.umn.edu/~geyer/mcmc/one.html` for an extended, informal and highly informative discussion by one of the first developers of MCMC in statistics, as well as to the *Handbook of Markov chain Monte Carlo* (Brooks et al., 2011) (Chapter 1: Introduction to Markov chain Monte Carlo; section 1.11.3: "One Long Run versus Many Short Runs").

We have hence focused our efforts on running two chains for a large number of iterations in order to efficiently use our hardware resources, over running a larger number of replicates for a smaller number of iterations. For the first replicate (file: BEAST_100m_run.1.log), all parameters and densities converge within the first 5 million iterations of the analysis, leaving over 95 million iterations (i.e. almost all of the run time) for the MCMC analysis to find a new part of the state space, which did not happen in this first replicate. For the second replicate (file: BEAST_100m_run.2.log), all parameters and densities converge within the first 10 million iterations of the analysis to the same values / densities of the first replicate, with hence 90 million iterations left for the MCMC analysis to find a new part of the state space, which again did not happen. Given the data set size, the fact that our replicates quickly converged to the same parameter values / densities, all continuous parameters yield high ESS values, and that the MCMC analyses had ample opportunity to reach different parts of the state space after the initial convergence, we consider these two replicates and the number of iterations for which they ran to prove sufficient evidence to not have to perform additional analyses.

8 Trace diagnostics

This section contains three examples of pathological traces and suggestions on how to solve them. Figure S8 shows a parameter trace (from a .log file generated by BEAST) that has not yet converged towards its posterior distribution. An upwards trend is clearly apparent throughout the entire trace plot, with no sign of the trace stabilizing or converging to a specific distribution of values. Hence, no suitable burn-in proportion can be chosen here and consequentially the ESS value for this parameter is far too low as indicated in red in the left Tracer panel (for all of the parameters in fact). This analysis had only run for a short time, i.e. approximately 3.5 million iterations, and hence the (initial) solution here is to run the analysis for much longer.

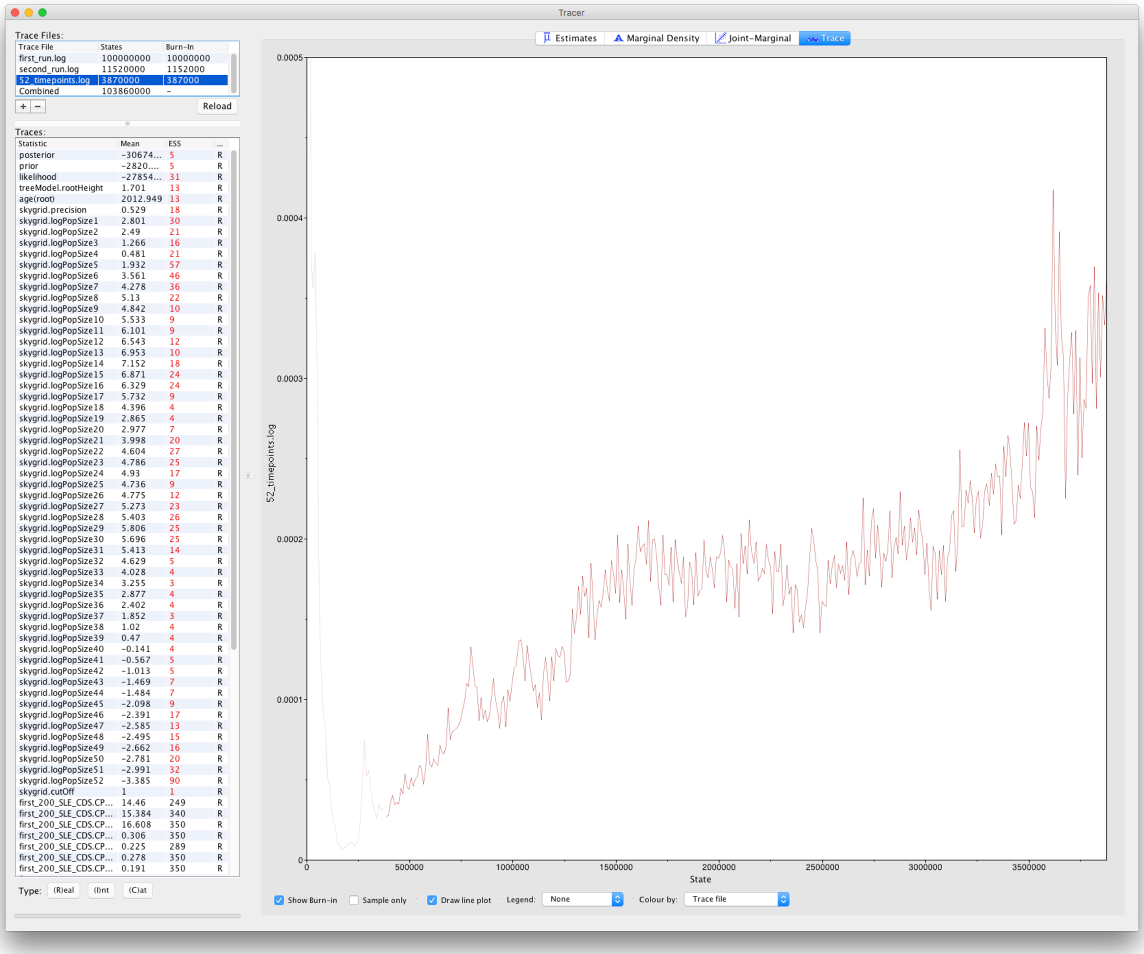

Figure S8: Tracer window showing an analysis that has not yet converged to the posterior distribution of one of its parameters as can be seen from the clearly still increasing trend in the right panel, as well as the low ESS values in the left panel.

Figure S9 shows a trace of the same analysis, but run for over 10 million iterations (rather than the 3.5 million iterations run before). Judging by the trace plot, it seems it takes the BEAST analysis roughly 4.5 million iterations to converge towards the posterior distribution for this particular parameter. The

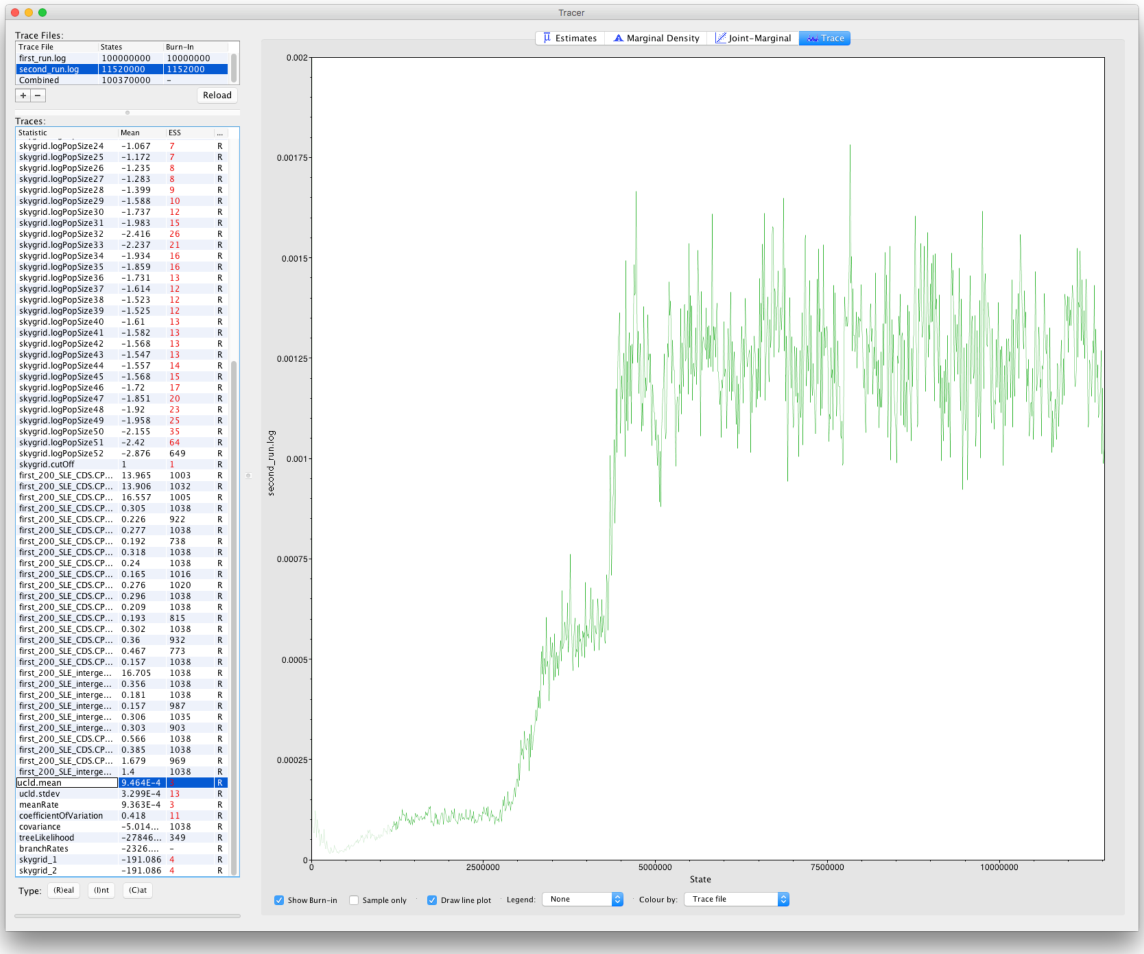

Figure S9: Trace plot of the mean clock rate parameter showing convergence towards its posterior distribution (although the analysis needs to be run for much longer to check whether another jump will occur in the trace plot, i.e. whether the analysis is momentarily stuck in a local optimum or not). Tracer's default burn-in of 10% is insufficient to reliably estimate the ESS value for this parameter and should hence be (manually) adjusted in the left panel.

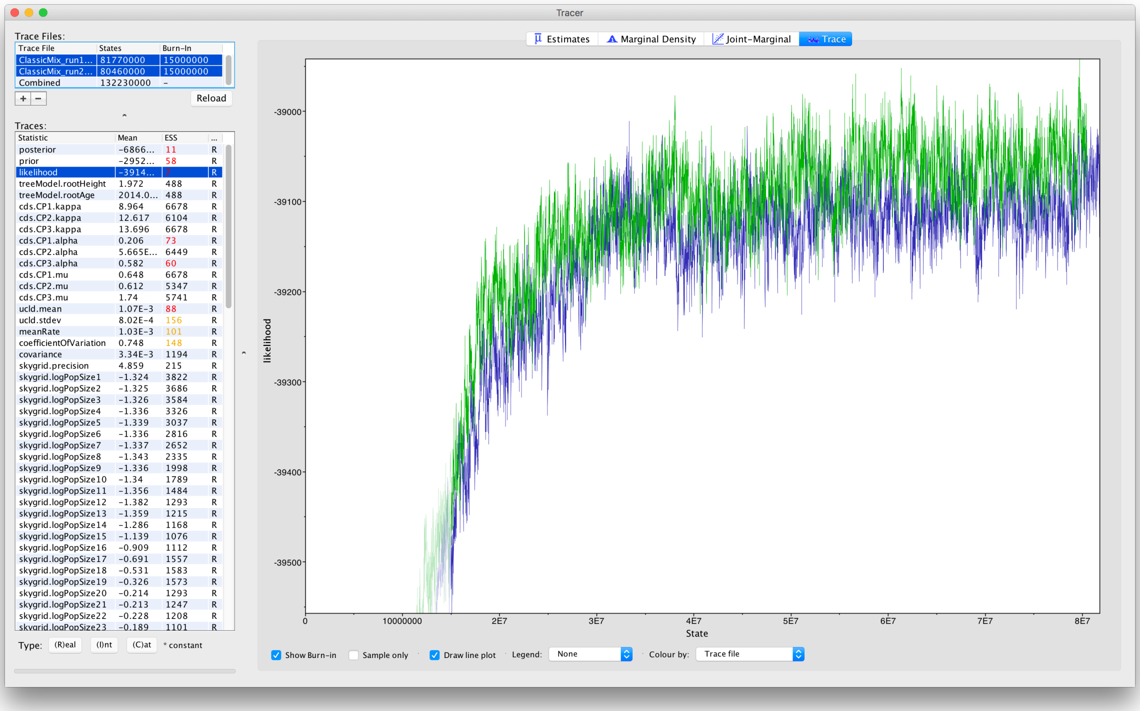

Figure S10: Tracer showing two independent BEAST runs (of the same XML) that have not (yet) converged to the same posterior distribution. Note that both traces are highlighted in the left panel so that they can be viewed on the same trace plot. Both traces can be seen distinctly and had not (yet) converged towards the same posterior distribution despite partially overlapping, although the blue trace at the end seems to be attempting a jump towards a higher posterior distribution region.

default burn-in of 10% of the run so far brings it at roughly 1.2 million iterations, which is insufficient here to obtain a reliable ESS value for this parameter. We can hence alter the burn-in for this analysis in the upper left panel of Tracer and set it to 4.5 million iterations and check the ESS value again. Note that this has to be checked for all the continuous parameters of the analysis and that the ESS values for these parameters need to be above 200 (see main text). The analysis shown here will therefore also need to be run for longer.

Figure S10 shows the traces of two independent BEAST runs of the same XML on a different data set than for the previous figures. When inspecting the likelihood trace of these two analyses individually, i.e. without plotting them in the same panel in Tracer, one could wrongfully pick the suboptimal trace to summarize the tree, reconstruct the population dynamics over time and report those results. Therefore, at least two independent replicates of the same analysis, using BEAST or any other Bayesian inference method/package, should be performed and carefully inspected and compared to one another. Importantly, whether or not the replicates have converted to the same posterior distribution, sufficient iterations need to be performed to ensure as best as possible that the analyses are not stuck in a local optimum and therefore still need to make one or more jumps towards higher posterior distribution regions.

9 Skygrid reconstruction and interpretation

In the main text, we focused on estimating the population dynamics over time using the non-parametric Skygrid coalescent model. The two independent BEAST replicates we performed in the previous section yield virtually identical Skygrid reconstructions, as can be seen in Figure S11.

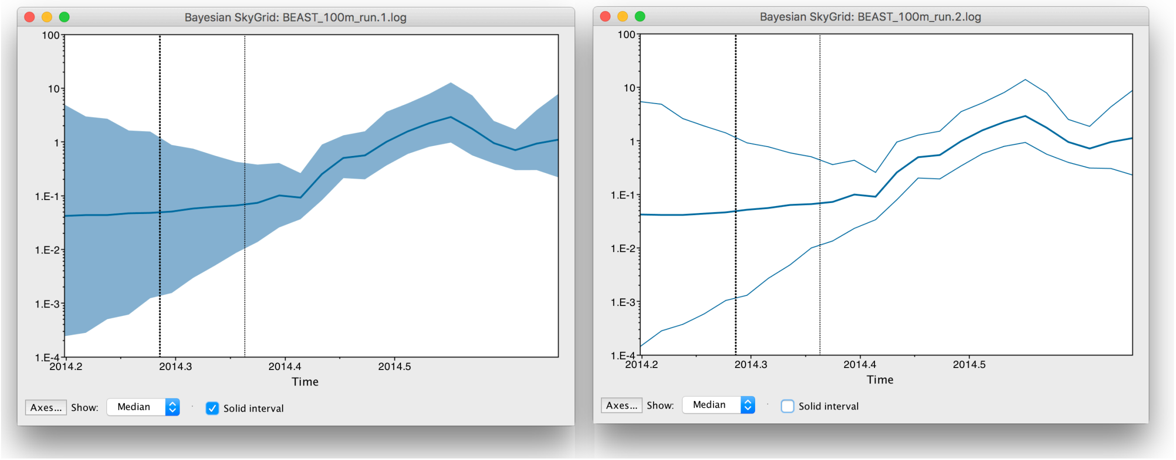

Figure S11: The Skygrid Reconstruction in Tracer visualises the estimated effective population sizes over time on a time axis. Shown here are the reconstructions obtained from two independent BEAST analyses, the one of the left plotted using a ‘Solid interval’ and the one on the right without this visualisation option.

However, it’s also important to check the obtained results against known observations, which may in an epidemic, for example, be available in the form of official case counts. In Figure S12, we again show the inferred effective population size over time, overlaid with the log-transformed number of new cases per week obtained from the WHO patient database for the same time period (note that the axes differ). The first official WHO situation report was released for the week commencing 19/05/2014, and so WHO case counts were not available before this point. As can clearly be seen, the Skygrid plot accurately mirrors the increase in case counts from mid-June 2014 onwards.

There are several aspects to consider when visualising these two quantities on top of each other. First, it is worth noting that despite the presence of negative values for the log of the effective population sizes in the Tracer output, there are no negative values in the Skygrid plot. We present non-log transformed effective population sizes in order to avoid having negative values in the Skygrid plot (as there are obviously no negative population sizes). It may hence be of greater interest to plot the log-transformed case counts on the log-transformed effective population sizes so that both quantities are in the log domain. This can be achieved using custom scripts, for example in the work of Dellicour et al. (2018).

Second, despite using different scales on the axes, it can be observed how well the estimated dynamics reflect reality, starting at small values (at the right-most dotted line) and quickly increasing over time. Notice that the increase in case counts is more jagged than the Skygrid. This is in part because of a GMRF smoothing prior on the effective population sizes being estimated, which smooths out the population size trajectory (Gill et al., 2013).

Finally, note that the estimated population sizes seem to stabilise towards the right of the plot while the case counts still increase slightly. This is a consequence of only selecting sequences from early on in the epidemic. Increasing effective population sizes are essentially a consequence of a rapid succession of coalescent events in the underlying phylogeny. As we reach the most recent samples in our data set, there are fewer coalescent events as there are fewer and fewer sequences to coalesce with one another. This therefore leads to an apparent plateauing in the effective population size estimates over time. Had

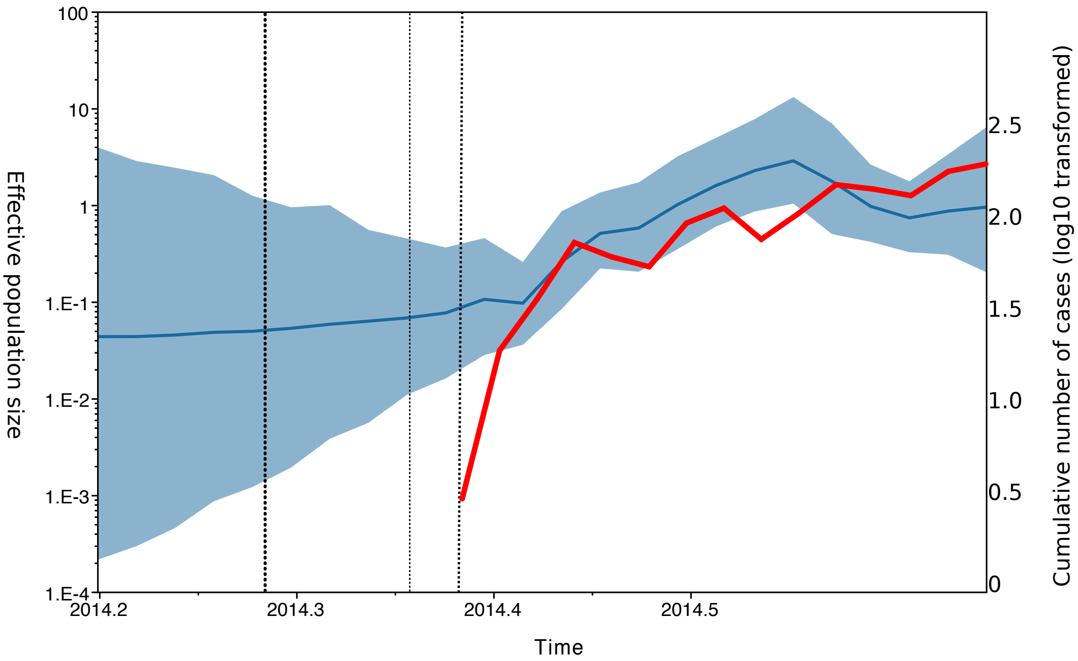

Figure S12: Skygrid analysis from the main text (the blue line is posterior median, and the shaded area is the accompanying 95% Bayesian credibility interval), with log-transformed case counts from the WHO patient database for the same time period overlaid in red. The furthest right dotted line shows the date (19/05/2014 or 2014.38) when the first official WHO case counts were available.

we continued to add more recent samples, we would expect to see increasing effective population size estimates, as in Figure 1e of Dellicour et al. (2018), which shows the fully reconstructed population dynamics throughout the whole 2013-2016 epidemic.

10 Ancient DNA analyses

Many of the analyses that focus on estimating population dynamics over time consider fast-evolving RNA viruses. When organisms evolve quickly, only a short range of sampling times is required for these analyses, e.g. in epidemic analyses where sampling times range from a couple of months to a few decades. For example, the earliest HIV-1 sequence/fragment that was used to reconstruct the early spread and epidemic ignition of HIV-1 in the Democratic Republic of Congo in the work of Faria et al. (2014) was sampled in 1959, with the evolutionary rate estimated at 3.26×10^{-3} substitutions per site per year.

However, these are not the only data sets that fit the definition of measurably evolving populations (MEPs), as these sorts of analyses can also be performed on more slowly-evolving molecular data, as long as there is ancient DNA present. This is because ancient DNA provides the drastically increased range of sampling times required to observe a sufficient number of mutations. A popular example of using mammalian data without any external calibration priors being used (i.e. only the genetic data and the sampling times of the sequences to calibrate the molecular clock) concerns the examination of population crashes in Beringian Steppe Bison (Shapiro et al., 2004). To investigate the evolution and demographic history of Pleistocene bison, the authors collected 442 bison fossils from Alaska, Canada, Siberia, China, and the lower 48 United States. Shapiro et al. (2004) used ancient DNA techniques to sequence a 685-base pair fragment of the mitochondrial control region, and accelerator mass spectrometry

radio-carbon dates were obtained for 220 samples, which spanned a period of over 60,000 years. By using such a wide sampling range, and despite the evolutionary rate being much slower than typically estimated in RNA virus data sets, at an estimated 3.20×10^{-7} substitutions per site per year, the resulting data set also constitutes an MEP. The authors were then able to reconstruct a detailed genetic history of bison throughout the late Pleistocene and Holocene epochs, which depicted a large diverse population living throughout Beringia until around 37,000 years before the present, when the population's genetic diversity began to decline dramatically. Similarly, Fu et al. (2013) use mitochondrial genome sequences from ten securely dated ancient modern humans spanning 40,000 years in order to yield a direct estimate of the mitochondrial substitution rate and date population splits in ancient human populations. The authors use a constant population size model and an uncorrelated relaxed clock model to thus estimate a substitution rate of 2.67×10^{-8} substitutions per site per year for the whole mitochondrial genome data set and 1.57×10^{-8} substitutions per site per year for the coding region of their data set. Ancient DNA sequences are hence able to offer valuable insights into molecular evolutionary processes which are not directly accessible via modern DNA (Ho et al., 2007).

The use of these methods is not limited to analysing mammalian data sets. For example, Ho et al. (2007) analyse ancient and modern DNA sequences from 19 species of animals, plants – containing a *Zea mays* sequence of approximately 4,500 years old – and bacteria. Finally, the study of larger DNA viruses can also exploit these techniques, as is shown in the work of Larsen et al. (2018). They were able to obtain two ancient papillomavirus (PV) sequences from rodents which were dated to be 27,000 and 23,000-years old, yielding an estimated evolutionary rate of 5.2×10^{-8} substitutions per site per year.

11 Main manuscript figures

This section contains full-size versions of the figures in the main text for reference.

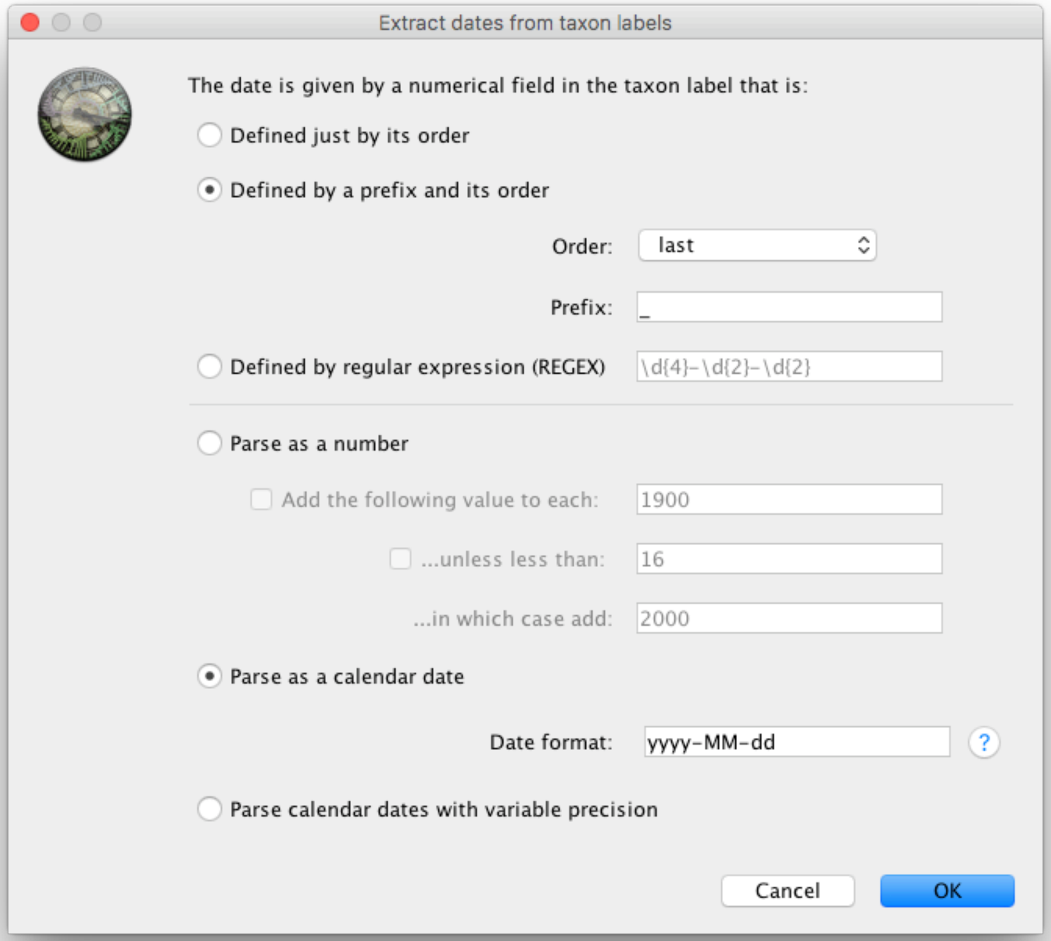

Figure S13: Larger version of Figure 2a in the main text: Dialog box showing how to extract sampling times from the sequence labels in TempEst.

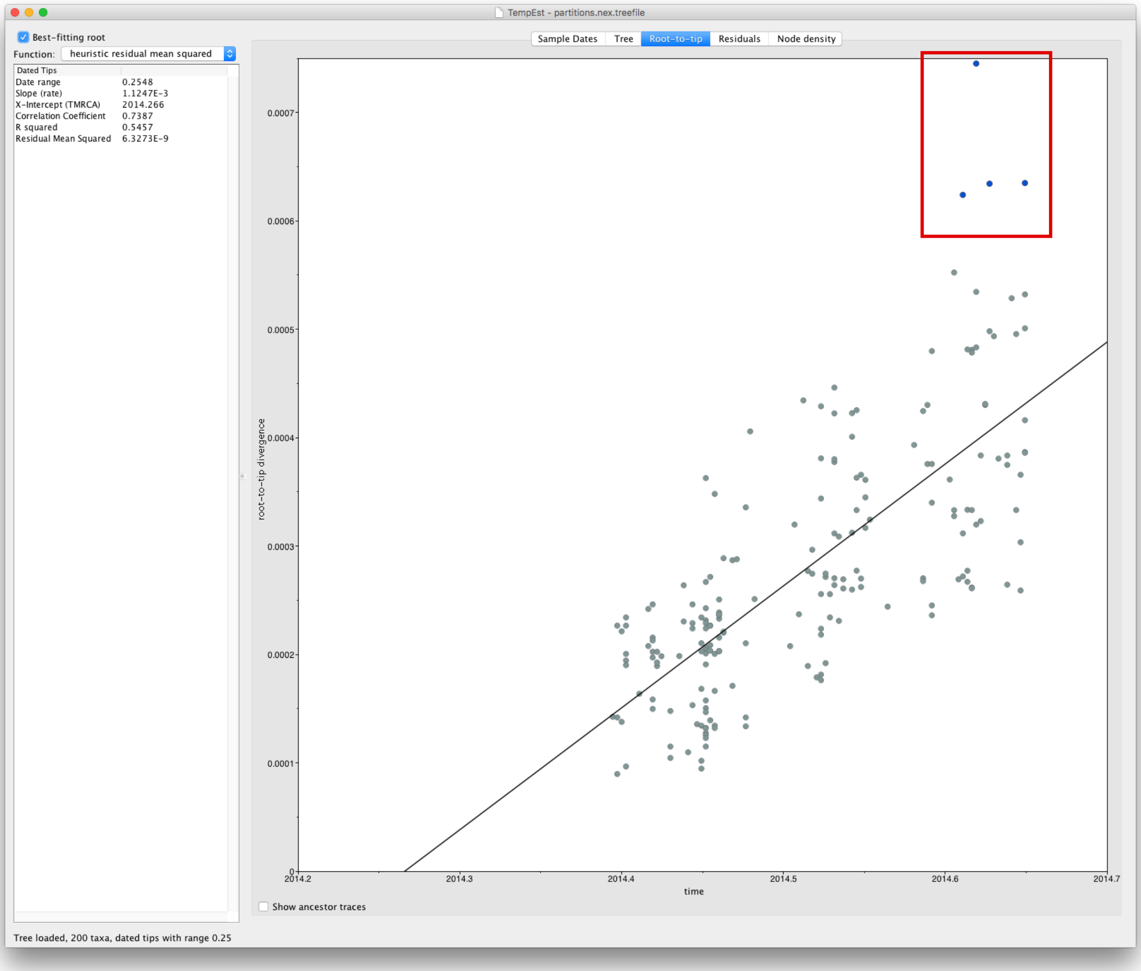

Figure S14: Larger version of Figure 2b in the main text: root-to-tip plot showing regression of genetic distance against time in TempEst. Four outliers can be identified and are indicated by the red box (which was manually added for illustration purposes and is not a feature of TempEst).

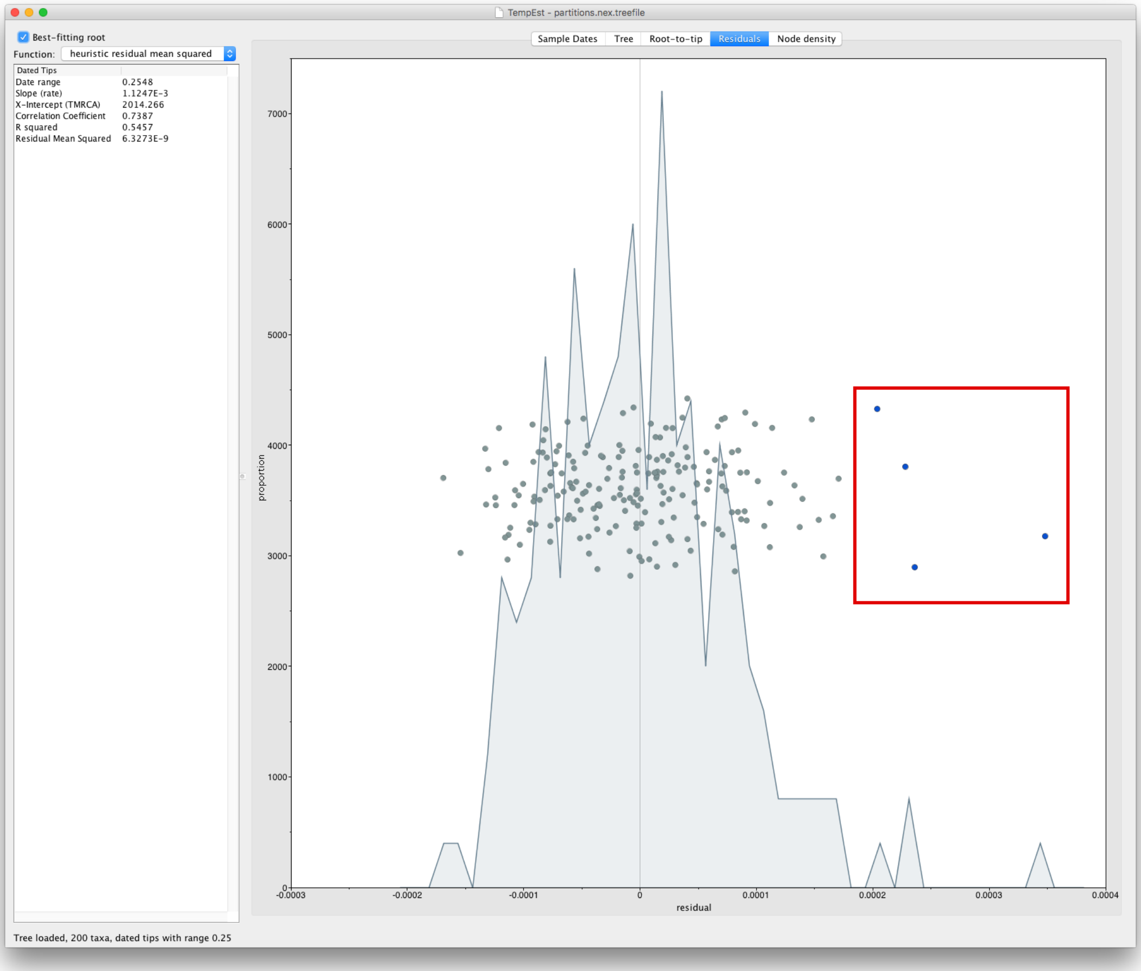

Figure S15: Larger version of Figure 2c in the main text: residuals plot in TempEst. Four outliers can be identified and are indicated by the red box (which was manually added for illustration purposes and is not a feature of TempEst).

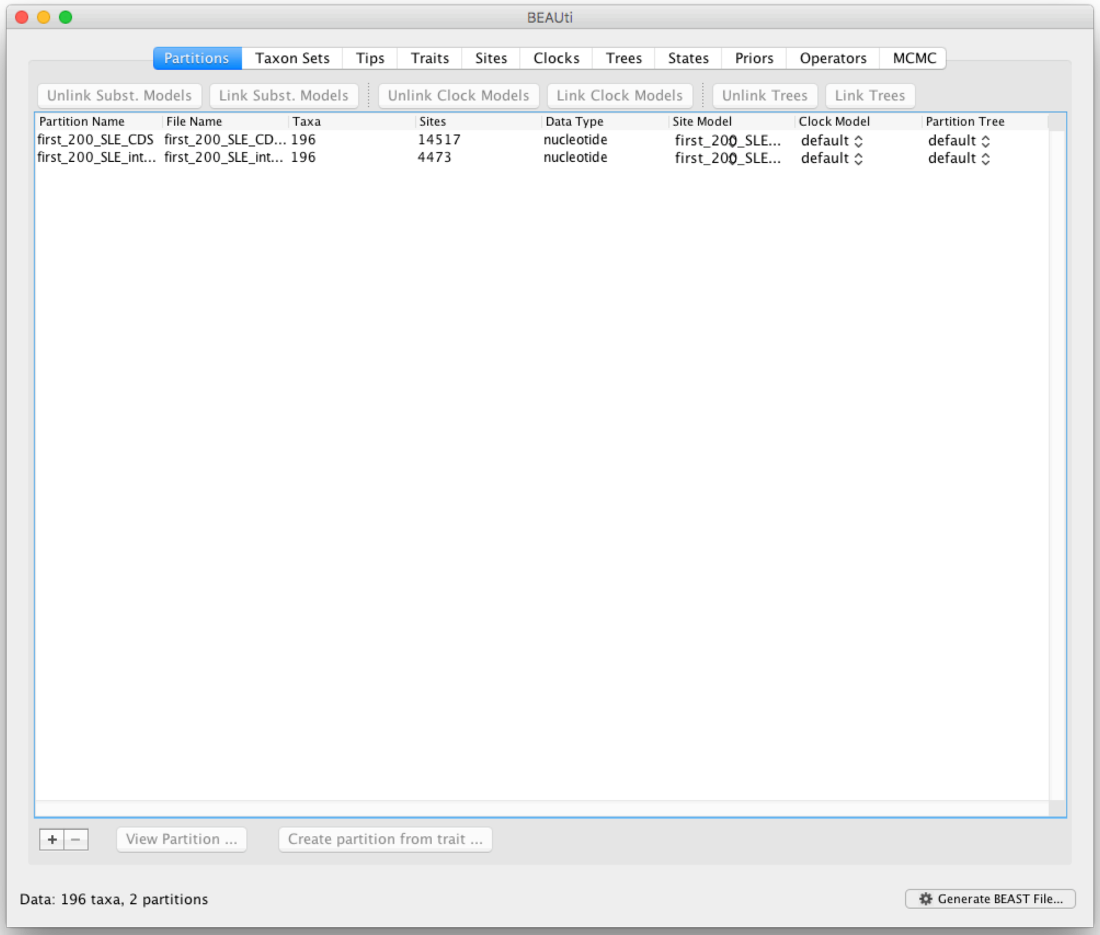

Figure S16: Larger version of Figure 3a in the main text: the data partitions we have imported using two different FASTA files for coding and intergenic regions in BEAUti.

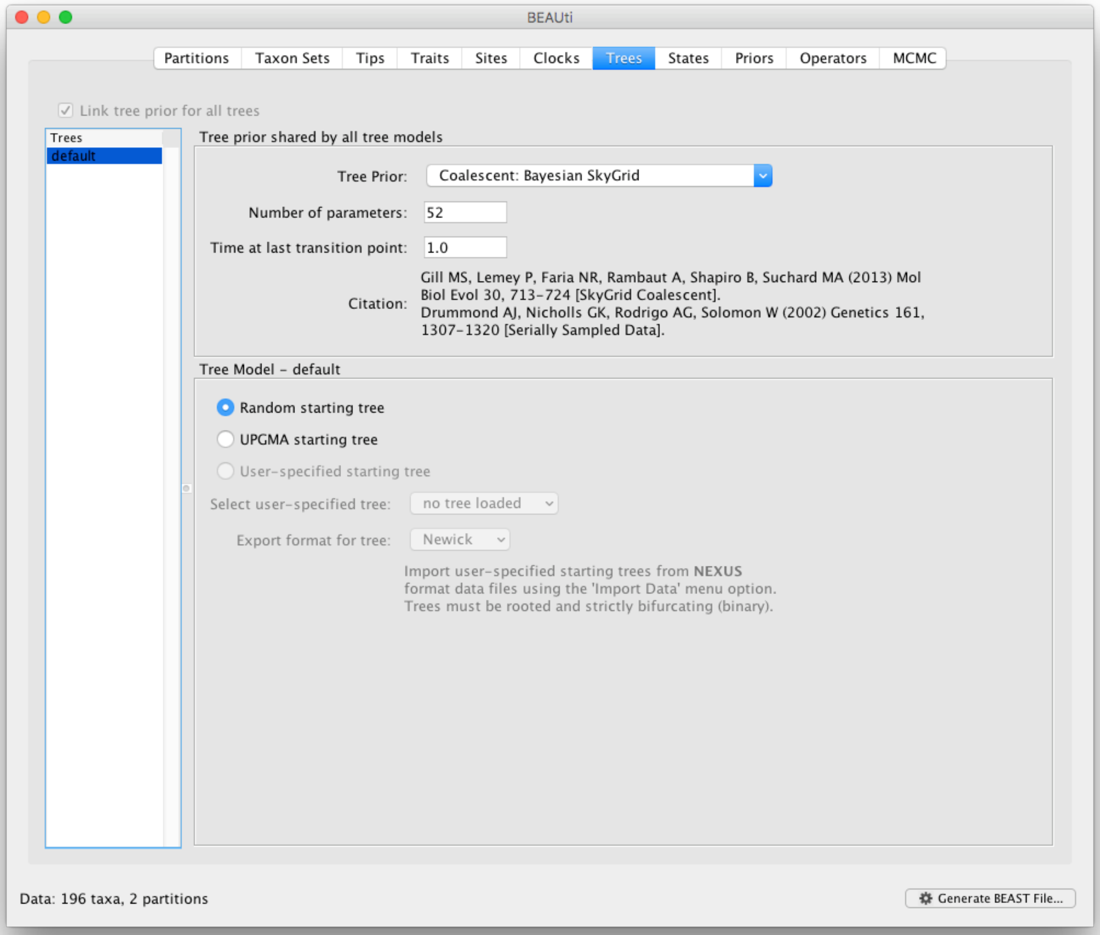

Figure S17: Larger version of Figure 3b in the main text: the **Trees** panel for setting up a Skygrid coalescent model to infer past population dynamics in BEAUti.

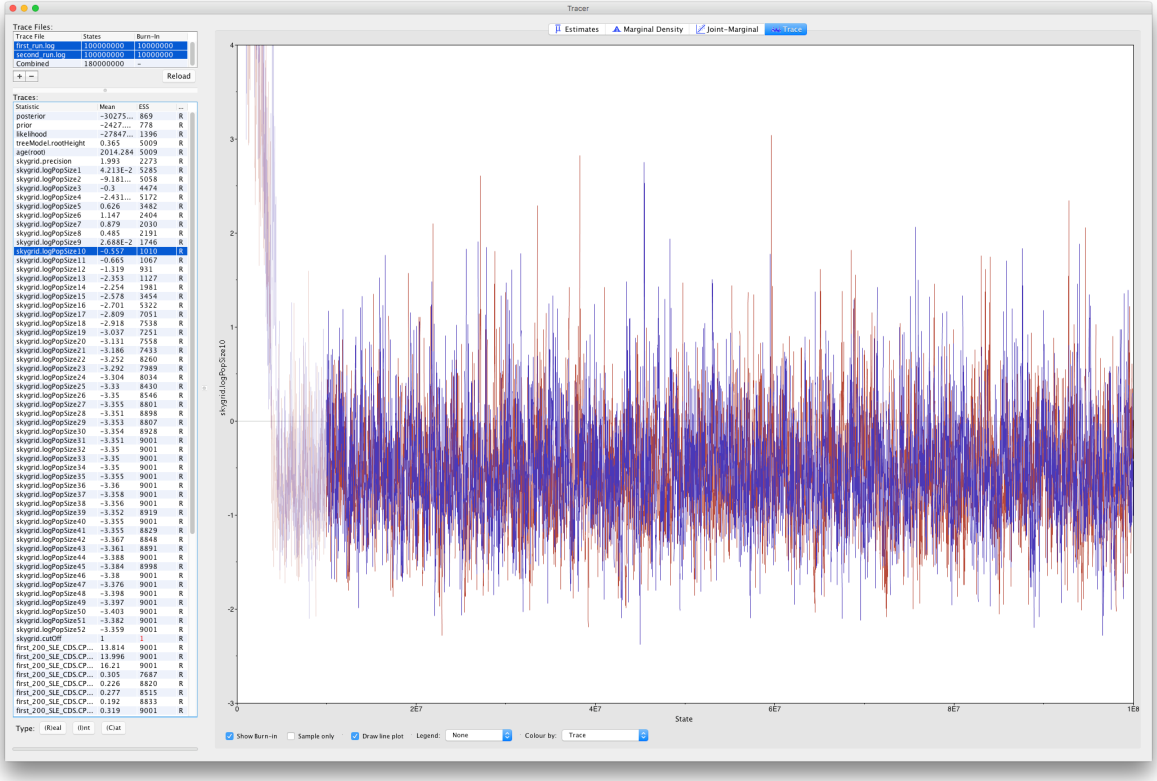

Figure S18: Larger version of Figure 4a in the main text: Tracer panel on the left shows the parameters logged during the run. Note that both runs are selected and as such, the panel on the right shows both traces in different colours, and the trace colours can be set using the **Colour by** dropdown menu.

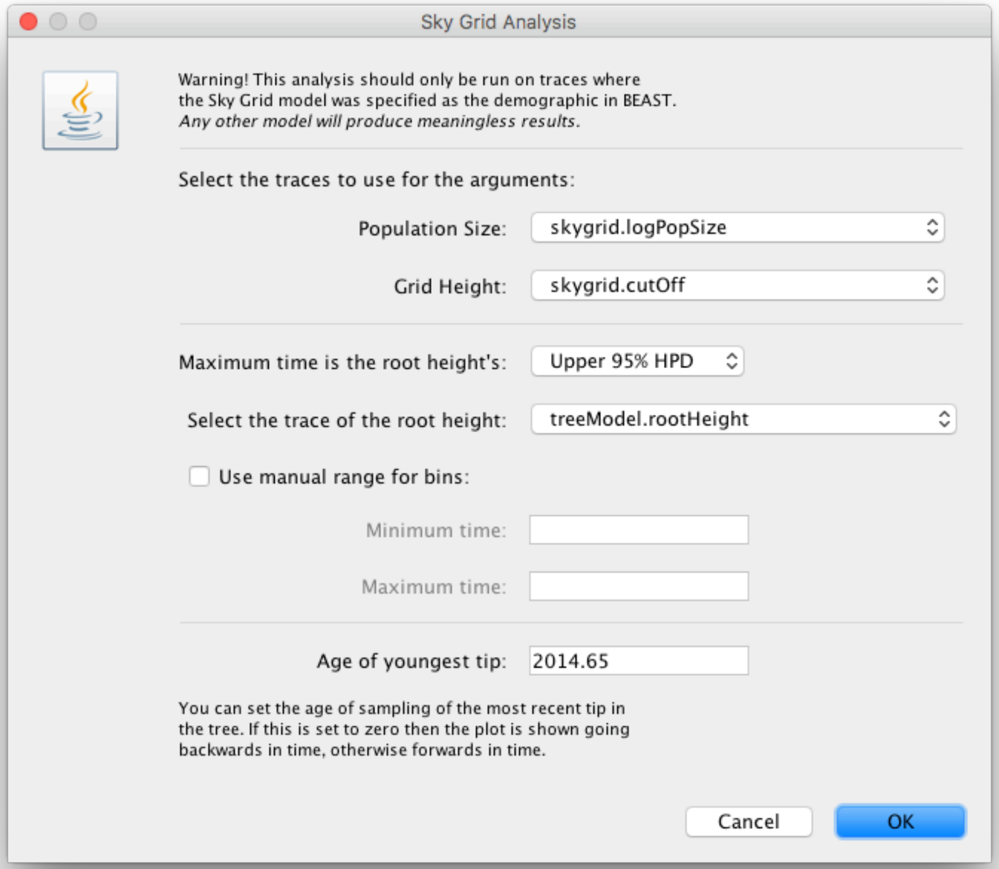

Figure S19: Larger version of Figure 4b in the main text: the options for the Skygrid reconstruction in Tracer based on the analysis in the main manuscript.

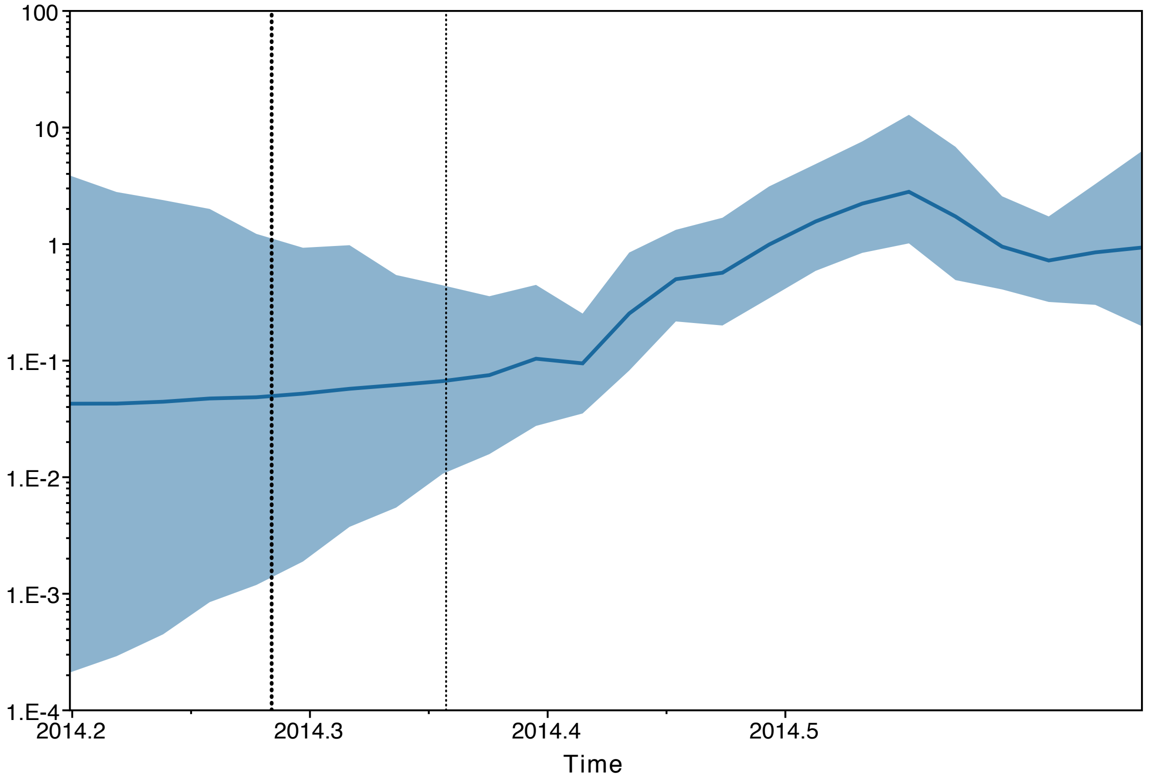

Figure S20: Larger version of Figure 4c in the main text: visualisation in Tracer of the past population dynamics using the Skygrid model. The shaded portion is the 95% Bayesian credibility interval (obtained by clicking the **Solid interval** checkbox in the lower left-hand corner of the visualisation window), and the solid line is the posterior median.

References

Ayres DL, Cummings MP, Baele G, Darling AE, Lewis PO, Swofford DL, Huelsenbeck JP, Lemey P, Rambaut A, Suchard MA. 2019. BEAGLE 3: improved performance, scaling and usability for a high-performance computing library for statistical phylogenetics. *Syst. Biol.* in press.

Brooks S, Gelman A, Jones G, Meng XL. 2011. Handbook of Markov chain Monte Carlo. Chapman and Hall/CRC.

Dellicour S, Baele G, Dudas G, Faria N, Pybus O, Suchard M, Rambaut A, Lemey P. 2018. Phylodynamic assessment of intervention strategies for the West African Ebola virus outbreak. *Nature Comms.* 9:2222.

Faria NR, Rambaut A, Suchard MA, et al. (14 co-authors). 2014. The early spread and epidemic ignition of HIV-1 in human populations. *Science.* 346:56–61.

Fu Q, Mittnik A, Johnson PLF, et al. (20 co-authors). 2013. A revised timescale for human evolution based on ancient mitochondrial genomes. *Curr. Biol.* 23:553–559.

Gill MS, Lemey P, Faria NR, Rambaut A, Shapiro B, Suchard MA. 2013. Improving Bayesian population dynamics inference: a coalescent-based model for multiple loci. *Mol. Biol. Evol.* 30:713–724.

Ho SYW, Kolokotronis SO, Allaby RG. 2007. Elevated substitution rates estimated from ancient DNA sequences. *Biology Letters.* 3:702–705.

Kumar S, Filipski AJ, Battistuzzi FU, Pond SLK, Tamura K. 2012. Statistics and truth in phylogenomics. *Mol. Biol. Evol.* 29:457–472.

Larsen BB, Cole KL, Worobey M. 2018. Ancient DNA provides evidence of 27,000-year-old papillomavirus infection and long-term codivergence with rodents. *Virus Evol.* 4.

Larsson A. 2014. Aliview: a fast and lightweight alignment viewer and editor for large datasets. *Bioinformatics.* 30:3276–3278.

Nakamura T, Tomii K, Yamada KD, Katoh K. 2018. Parallelization of MAFFT for large-scale multiple sequence alignments. *Bioinformatics.* 34:2490–2492.

Nguyen LT, Schmidt HA, von Haeseler A, Minh BQ. 2014. IQ-TREE: A fast and effective stochastic algorithm for estimating maximum-likelihood phylogenies. *Mol. Biol. Evol.* 32:268–274.

Rambaut A, Drummond AJ, Xie D, Baele G, Suchard MA. 2018. Posterior summarization in Bayesian phylogenetics using Tracer 1.7. *Syst. Biol.* 67:901–904.

Rambaut A, Lam TT, Max Carvalho L, Pybus OG. 2016. Exploring the temporal structure of heterochronous sequences using TempEst (formerly Path-O-Gen). *Virus Evol.* 2:vew007.

Sagulenko P, Puller V, Neher R. 2018. TreeTime: maximum-likelihood phylodynamic analysis. *Virus Evol.* 4:vex042.

Shapiro B, Drummond AJ, Rambaut A, et al. (27 co-authors). 2004. Rise and fall of the Beringian steppe bison. *Science.* 306:1561–1565.

Suchard M, Lemey P, Baele G, Ayres D, Drummond A, Rambaut A. 2018. Bayesian phylogenetic and phylodynamic data integration using BEAST 1.10. *Virus Evol.* 4:vey016.

To TH, Jung M, Lycett S, Gascuel O. 2015. Fast Dating Using Least-Squares Criteria and Algorithms. *Syst. Biol.* 65:82–97.

Volz EM, Frost SDW. 2017. Scalable relaxed clock phylogenetic dating. *Virus Evol.* 3.
